# Circular RNA hsa-circ-0005238 enhances trophoblast migration, invasion and suppresses apoptosis *via* the miR-370-3p/CDC25B axis

**DOI:** 10.3389/fmed.2022.943885

**Published:** 2022-10-13

**Authors:** Zhuomin Huang, Litong Zhu, Quanfu Zhang, Depeng Zhao, Jilong Yao

**Affiliations:** ^1^Shenzhen Maternity and Child Healthcare Hospital, The First School of Clinical Medicine, Southern Medical University, Shenzhen, Guangdong, China; ^2^Department of Gynecology, Affiliated Shenzhen Maternity and Child Healthcare Hospital, Southern Medical University, Shenzhen, Guangdong, China; ^3^Shenzhen Baoan Maternal and Child Health Hospital, Jinan University, Shenzhen, Guangdong, China; ^4^Department of Reproductive Medicine, Affiliated Shenzhen Maternity and Child Healthcare Hospital, Southern Medical University, Shenzhen, Guangdong, China

**Keywords:** circular RNAs, fetal growth restriction (FGR), hsa-circ-0005238, trophoblast, miR-370-3p

## Abstract

**Background:**

Fetal growth restriction (FGR) is attributed to various maternal, fetal, and placental factors. Trophoblasts participate in the establishment and maintenance of pregnancy from implantation and placentation to providing nutrition to fetus. Studies have reported that impaired trophoblast invasion and proliferation are among factors driving development of FGR. Circular RNAs (circRNAs) can regulate trophoblast function. We assessed the significance of circRNAs underlying FGR development.

**Materials and methods:**

Next generation sequencing (NGS) was carried out to quantify levels of circRNAs in placenta tissues with and without FGR. *In vitro* experiments including transfection, (3-(4,5-dimethylthiazol-2-yl)-5-(3-carboxymethoxyphenyl)-2-(4-sulfophenyl)-2Htetrazolium) (MTS) assays, flow cytometry analyses, Transwell assays, wound healing assays, western blotting, qRT-PCR, dual-luciferase assays, immunofluorescence staining, and RIP assay were performed.

**Results:**

There were 18 differentially expressed circRNAs between FGR placentas and uncomplicated pregnancies, while levels of hsa-circ-0005238 were markedly low in FGR placentas. Our *in vitro* experiments further revealed that hsa-circ-0005238 suppressed apoptosis and enhanced proliferation, migration, invasion of trophoblast cell lines. The hsa-miR-370-3p was identified as a direct target of hsa-circ-0005238. Mechanistically, hsa-miR-370-3p prevents invasion as well as migration of trophoblast cells by downregulating CDC25B.

**Conclusion:**

The hsa-circ-0005238 modulates FGR pathogenesis by inhibiting trophoblast cell invasion and migration through sponging hsa-miR-370-3p. Hence, targeting this circRNA may be an attractive strategy for FGR treatment.

## Introduction

Fetal growth restriction (FGR) is a pathological condition characterized by an estimated fetal weight or abdominal circumference of less than the 10th percentile for gestational age (7). FGR is the significant risk factor of neonatal death, accounting for 5–10% of newborns, increasing the risk for mortality by 2-fold compared to those with normal fetal growth (1, 2). Moreover, growth restriction of fetuses also contributes to the development of diabetes, cardiovascular disease, and stroke in adulthood (3–5).

The known risk factors of FGR include maternal complications (including pre-eclampsia, connective tissue disorders, and type 2 diabetes) and fetal complications (such as twin pregnancies, aneuploidy), accounting for 70% of FGR cases (1, 6). The etiology of FGR is complex, despite uterine–placental perfusion is considered as a major cause (7), mechanism underlying the pathogenesis of FGR remains unclear.

Dysregulated non-coding RNAs (ncRNAs) is association with initiation as well as progression of many diseases (8–10), and the FGR is not an exception. As single-stranded RNAs, CircRNAs can form covalently closed loops and have multiple roles, including parental gene expression regulation, direct interactions with RNA-binding proteins, and sponging miRNAs (11, 12). CircRNA studies are mainly lying in the field of tumors and other chronic ailments, and little is known about placental transcriptome, especially in disease settings. Recently Gong et al. (13) analyzed human the transcriptome of 302 placentas from humans, including 56 FGR cases, and characterized several dysregulated transcripts relating to FGR. They also found some placental circular RNAs may potential be diagnostic or therapeutic targets of FGR. In another study, Medina-Bastidas et al. (14) also reported 134 lncRNAs were differentially expressed in the FGR placentas compared to normal ones, evidencing the potentially critical role of the lncRNA as part of the pathophysiology of this disease. However, few studies have mechanistically investigated how the ncRNAs regulate the trophoblasts in FGR, which prevents unraveling their definitive role in the pathogenesis of FGR. As a result, further studies in depth are required to investigate the significance of circRNAs in FGR.

We first used next generation sequencing (NGS) to investigate the transcriptome profiles of placentas healthy and FGR pregnant women. Then, we verified the differentially expressed circRNAs using quantitative real-time polymerase chain reaction (qRT-PCR). Furthermore, we evaluated the significance of identified hsa-circ-0005238 *via* regulating the miR-370-3p/CDC25B axis in biological process in cultured trophoblast cells. Hence, we determined the mechanisms of pathogenesis in FGR.

## Materials and methods

### Tissue samples

Forty placentae from FGR pregnant women and forty placentae from normal pregnant women were included for analyses. FGR was defined as an estimated fetal weight or abdominal circumference of less than the 10th percentile. The participants of both groups were singleton pregnancy and between 20 and 40 of age. All participants had normal blood routine, normal blood pressure, normal liver and kidney function, and normal blood glucose levels, as well as without other pregnancy complications. Three placenta samples of FGR and three of normal placenta were collected for circRNA NGS. All samples were for qRT-PCR assay. Each placental tissue was divided into four parts. One part of the placental tissues was immediately immersed in RNA protective solution (Sangon Biotech, Shanghai, China) after collection, thereafter frozen in liquid nitrogen for use later. The Medical Ethics Committee of Shenzhen Maternity and Child Healthcare Hospital approved the study ([2018]280). Written informed consents were obtained from all participants.

### RNA sequencing

Three placental samples of both groups (FGR and normal control) were used for RNA sequencing. The TRIzol reagent (Invitrogen, Carlsbad, CA, USA) was used for extraction of total RNA from tissues. The Agilent Bioanalyzer 2,100 system (Agilent Technologies, CA, USA) was used for assessment of RNA integrity. Total RNA samples were used in subsequent experiments if the following requirements were met: RNA integrity number (RIN) ≥ 7.0, OD_260/280_ ≥ 1.7 and 28S:18S ratio ≥ 1.5:1. Library construction and RNA sequencing service were provided by CapitalBio Co., Ltd. (Beijing, China). Five micrograms RNA per sample was used. Base calling was performed on the original image files of Illumina high-throughput sequencing, which was converted into raw data. Sequencing libraries were generated using the rRNA-depleted and RNase R digested RNAs by NEBNext^®^ Ultra™ Directional RNA Library Prep Kit for Illumina^®^ (NEB, USA) following manufacturer’s recommendations. The library sequencing was sequenced on Novaseq6000 (Illumina, USA). FastQC software was applied to evaluate raw data sequencing quality and the content of A, T, C, and G bases in the sequencing data. Low quality data were filtered using fastp software. Tophat2 software was used for aligning clean reads with high quality and the human reference genome. Find_circ and CIRCexplorer2 software (15) was then used to subject these sequencing data which could not be aligned to reference genome to subsequent circRNA analysis *via* reverse splicing event recognition. Differentially expressed circRNAs were analyzed by the SRPBM software (16). A customized R software package was used for quantile normalization. CircRNAs with | log2FC| ≥ 1 and *p* ≤ 0.05 were markedly differentially expressed as determined by Limma package of R software and edge package of R software. KOBAS software (17) was conducted for analysis of pathway enrichment and functional annotation for host genes of differentially expressed circRNAs.

### RNA extraction and reverse transcription-quantitative PCR assay

Total RNA from placental tissues or trophoblast cells was isolated with TRIzol reagent (Invitrogen). The ImProm-IITM Reverse Transcription System (Promega, Madison, WI, United States) was adopted to produce cDNA from RNA. Reverse transcription used random primers to detect circRNAs. RT-qPCR assay was conducted in the SYBR GREEN qPCR Super Mix (Promega, United States). The thermal cycling conditions used in the RT-qPCR reaction were 95^°^C for 5 min, followed by 40 cycling of 95^°^C for 15 s and 60^°^C for 32 s. Expressions of GAPDH were opted as internal controls for target circRNAs and mRNAs. The expression of U6 functioned as internal control for miRNAs. Assays were conducted in triplicates. Relative levels of RNA were quantified using 2^–ΔΔ*Ct*^ values. Primers for RT-qPCR assay were present in Table 1.

**TABLE 1:** Primer of circular RNAs (circRNAs).

CircRNA	Sequence	Size of product
Hsa_circ_0006427-F	GCCAGGCTGTGACTTTTGAA	156bp
Hsa_circ_0006427-R	TAACCTTGTGATCTGCCAGGA	
Hsa_circ_0103279-F	AGTGCTAAAAAGGAGAAATTGTCG	196bp
Hsa_circ_0103279-R	AAAGGGACATCGTGTTCACC	
Hsa_circ_0005238-F	AGTTTTGCAGTCGAGGAGTT	143bp
Hsa_circ_0005238-R	GCAAGTTGTGCATCCCATTC	
Hsa_circ_0035897-F	TCCTCAGTGCTTGGTCCATC	150bp
Hsa_circ_0035897-R	TTAGTGGCGTCACAGAATCAG	
Hsa_circ_0072697-F	AGGCTTATCCAACCAGCGTA	201bp
Hsa_circ_0072697-R	TGCATGTAACTGCGCTCATA	
Hsa_circ_0000972-F	GCTGATTTTTCAGGGTCAACT	131bp
Hsa_circ_0000972-R	GCCCAAAAGGGTAGCAACTA	
Hsa_circ_0088213-F	TCGCCAGAATGCACTGTTAC	171bp
Hsa_circ_0088213-R	CATCCAAGTAGGCTCTTTCAA	
Hsa_circ_0084748-F	TCTGGCATCCATTTGCGTTA	235bp
Hsa_circ_0084748-R	CGCCACTAGCATGTAGAAGAA	
Hsa_circ_0137008-F	TGGCAGTCTCACATTGAAGG	316bp
Hsa_circ_0137008-R	CCTTCCAAGGGAACATCTGTG	
Hsa_circ_0006222-F	ACAGCAGAAGAGATGATATTGCT	136bp
Hsa_circ_0006222-R	TCAGAAAGCGAAAGCTGGTC	
Hsa_circ_0005939-F	ACCCTTCTCAATCATCAGCAA	167bp
Hsa_circ_0005939-R	TTGTTCATCTGATTTTCTCTTGT	
Hsa_circ_0005286-F	CATCAGAACAGAGTGGTGCT	147bp
Hsa_circ_0005286-R	GGCACAATAGTTGTAGAGGCA	
Hsa_circ_0007440-F	ACATGGGCAATGTGATTTGATG	216bp
Hsa_circ_0007440-R	TTGCCAGCATTCCTCAACTT	
Hsa_circ_0005078-F	AGATGTTCTTGCACAGGGTG	137bp
Hsa_circ_0005078-R	GACAGGTTGTCTGTATACTGCT	
Hsa_circ_0003288-F	ACAGGTTGGTCCTACAGC	168bp
Hsa_circ_0003288-R	AGTTGGCCTCGTGGCATT	
Hsa_circ_0002590-F	CTCCGCACGGTATTATTG	157bp
Hsa_circ_0002590-R	GTCCTGCTATTTCTCCTCTT	
Hsa_circ_0086190-F	GACACGCCCGCACAAAGA	108bp
Hsa_circ_0086190-R	GCAATGCTGGCAAACAGG	
Hsa_circ_0005204-F	GTTCTACTCCTCCGTCTTCG	254bp
Hsa_circ_0005204-R	CTCCAGCATCTTGTTCACAG	

### Cell cultures and transfection

The extra-villous trophoblast cell line (HTR-8/SVneo) of humans from the American Type Culture Collection (ATCC) was employed for *in vitro* experiments. The HTR-8/SVneo cells were grown in RPMI 1640 medium (Gibco, Carlsbad, CA, United States) added with penicillin/streptomycin (1%, Solarbio, Beijing, China) and 10% fetal bovine serum (Gibco, Carlsbad, CA, United States). HTR-8/SVneo cells were incubated in humidified 5% CO_2_ air at 37^°^C.

Overexpression vector for hsa-circ-0005238 (ov-circ-0005238) was constructed by Guangzhou Geneseed Biotech Co., Ltd., China. Negative control (NC) was empty pLCD5H-ciRplasmid. Small interfering RNA (siRNA) binding hsa-circ-0005238 was synthesized to knockdown hsa-circ-0005238. HTR-8 cells (5 × 10^4^ cells/well) were seeded in six-well plates and grown overnight. The above vector (1 μg/per well) or siRNAs (50 nm) was transfected into HTR-8/SVneo cells using 10 μl/per well Lipofectamine TM 2,000 Reagent (Invitrogen, Carlsbad, CA, United States). MiR-370-3p mimics (50 nm), inhibitors (50 nm) and their matching negative controls (miR-NC and miR-NC-inhibitor) were obtained from GenePharma Co. (Shanghai, China). Lipofectamine 2000 reagent (10 μl/per well) was used for cellular transfections. After transfection, cells were collected for the subsequent assays.

### Cell proliferation analyses

(3-(4,5-dimethylthiazol-2-yl)-5-(3-carboxymethoxyphenyl) -2-(4-sulfophenyl)-2H-tetrazolium) (MTS) assay for cell proliferation capability was conducted using the CellTiter 96 Aqueous One Solution Cell Proliferation Assay Kit (Promega, Madison, WI, USA). HTR-8/SVneo cells were seeded onto 96-well plates after transfections. After the cells were incubated with MTS solution for 4 h, the absorbances were measured at 490 nm by Microplate Reader (Thermo Fisher Scientific, MA, United States).

### Cell apoptosis analysis

Flow cytometry for cell apoptosis was conducted using standard protocols of Annexin V-FITC/PIApoptosis Detection Kit (KeyGEN Biotech, Nanjing, China). HTR-8/SVneo cells were harvested 48 h after transfections and further incubated for 5 min on ice away from light with a mixture of 5 ml propidium iodide and 5 ml Annexin V-FITC. BD FACSCalibur flow cytometer was used for analyses of HTR-8/SVneo cell apoptosis. Cell apoptosis index was demonstrated in the FlowJo software version 8 (FlowJo, Ashland, OR, United States). Early and late (+) apoptotic cells were stained by AnnexinV-FITC^+^/PI^–^ or AnnexinV-FITC^+^/PI^+^, respectively. Cell apoptosis proportion was determined by the counts of late or early cells over the total counts of all apoptotic cells. Each experiment was conducted in triplicates.

### Cell migration and invasion analyses

Twenty four hours post transfection, the HTR-8/SVneo cells were grown in serum-free medium for starvation of 24 h. Migration assay was performed in 24 well and 8 μm pore Transwell chambers (Corning, Steuben County, NY, United States). The invasion experiments were done in Transwell chambers with Matrigel (BD Biosciences, Bedford, MA, United States). HTR-8/SVneo cells moving to the lower membrane surface were counted.

### Wound healing assay

The transfected HTR-8/SVneo cells were inoculated into a six-well plate at 37^°^C until 90% confluent 24 h after transfection. Subsequently, a 10 μl pipette tip was used to make an injury line. Following culturing with serum-free medium at 37^°^C, images of the migrated cells were captured at 0, 6, 24, and 48 h by microscope (Olympus Corporation, Japan).

### Bioinformatics analyses

All circRNAs information was acquired from circBase.^1^ Target miRNAs of hsa-circ-0005238 or hsa-miR-370-3p were searched the TargetScan databases.^2^

### Dual luciferase activity assays

Wild-type hsa-circ-0005238 fragments, including binding sequences for hsa-miR-370-3p were amplified and there after cloned into psi-CHECK-2 vector (Promega) to assemble the recombinant luciferase reporter plasmid. This plamid was termed as “wt-circ-0005238.” In this recombinant plasmid, the binding sequences of hsa-miR-370-3p were mutated *via* site-directed mutagenesis using one-step overlap extension PCR. This mutated plasmid was termed as “mut-circ-0005238.” HTR-8/SVneo cells were further transfected with 100 ng recombinant plasmid and 50 nm of hsa-miR-370-3p mimic or miR-NC on 24-well plates. At 48 h post co-transfection, Dual-Luciferase Reporter Assay System (Promega) was adopted to assess Firefly and Renilla luciferase activities as instructed by the manufacturer. Relative luciferase activities were expressed as Renilla and Firefly luciferase activities ratios (R/F). Assays were conducted in triplicate.

### Anti-AGO2 RNA immunoprecipitation assay

This assay was conducted using the Magna RIP RNA-Binding Protein Immunoprecipitation Kit instructions (Millipore, Bedford, MA, United States). Briefly, HTR-8/SVneo cells transfected with hsa-miR-370-3p mimic or miR-NC were prepared and then were lysed in polysome lysis buffer with protease inhibitor cocktail. Before immunoprecipitation, a positive control in the RIP assay, called “input,” was obtained from the cell lysate. Following that, cell lysate (100 μl) was treated overnight at 4^°^C in the presence of a magnetic bead-antibody (IgG or Ago2). Finally, the RNA was isolated and purified. Hsa-circ-0005238 levels in purified RNA were evaluated by RT-qPCR. Each experiment was conducted in triplicates.

### Western blot assays

Protein levels were assessed by the Bio-Rad Protein Assay Kit (Bio-Rad Laboratories, Hercules, CA, United States). To separate proteins, sodium dodecyl sulfate-polyacrylamide gel electrophoresis was used. Then, proteins were transferred onto polyvinylidene fluoride membranes that were treated overnight at 4^°^C with primary antibodies (CDC25B, 1:1,000. Abcam, Cambridge, MA, United States), washed and incubated for 2 h at room temperature (RT) with secondary antibodies (anti-rabbit IgG, HRP-linked Antibody, 1:5,000). Lastly, increased chemiluminescence (Pierce, Rockford, IL, United States) was used to see the bands on the membranes, and the signals were subjected to X-ray. Each experiment was performed in triplicates. The ratios of target protein densitometric values to GAPDH densitometric values were used to measure relative protein expression.

### Statistical analysis

Data were shown as mean ± SD. *T*-tests or non-parametric tests were employed to analyze the data of normal or skewed distribution. *P* < 0.05 denoted significance. GraphPad Prism version 5.0 (GraphPad Software, San Diego, CA, United States) and SPSS version 20.0 (IBM Corp, Armonk, NY, United States) were used to analyze the data.

## Results

### The clinical features of fetal growth restriction and healthy pregnant women

Table 2 shows that there were no marked differences in maternal age, height, weight, BMI, and week of delivery in the FGR group and matched normal control group (*P* > 0.05), while birth weight of the fetus, fetal length, fetal head circumference, and fetal thoracic circumference in the FGR group were markedly low relative to those in matched normal control group (*P* < 0.001).

**TABLE 2:** Summary of clinical characteristics.

Items	FGR	Control	*P*-value
Number	40	40	
Age (years)	29.08 ± 3.25	28.35 ± 3.83	0.847
Height (cm)	159.59 ± 5.26	159.81 ± 5.13	0.364
Weight (Kg)	60.70 ± 9.04	63.356 ± 6.27	0.131
BMI	23.78 ± 3.10	24.77 ± 1.69	0.080
Week of delivery	37.55 ± 0.71	37.75 ± 0.67	0.200
Birth weight of the fetus (g)	2285.50 ± 192.83	3254.50 ± 289.88	*p* < 0.001
Fetal length (cm)	46.75 ± 2.33	50.10 ± 0.71	*p* < 0.001
Fetal head circumference (cm)	32.35 ± 1.10	34.13 ± 0.69	*p* < 0.001
Fetal thoracic circumference (cm)	31.35 ± 1.10	33.13 ± 0.69	*p* < 0.001

### Differentially expressed circular RNAs and cluster analysis

**FIGURE 1:**
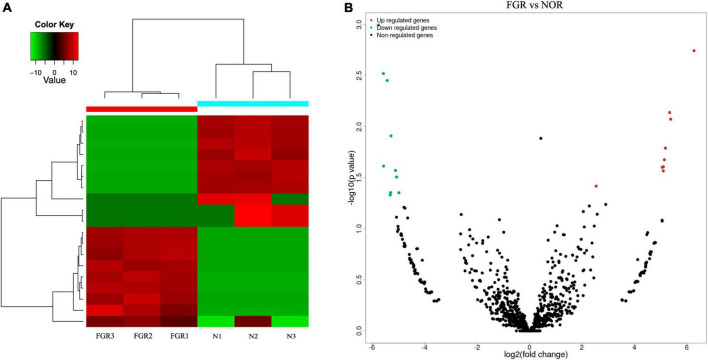
Bioinformatic analysis of the results of circRNA. **(A)** Heatmap of differentially expressed circRNAs between groups. **(B)** Volcano plots of differentially expressed circRNAs between groups. Red: up-regulated; green: down-regulated.

Next generation sequencing of three placenta samples from each group was conducted to assess the significance of placenta abnormalities in FGR pathogenesis. RNA sequencing data analyzed in the article were deposited into the CNGB Sequence Archive (CNSA) (18) of the China National GeneBank DataBase (CNGBdb) (19)^3^ under accession number CNP 0002703. There were 3,069 differentially expressed circRNAs between the groups. After applying a criterion of *P* ≤ 0.05 and | log2FC| ≥ 1, 19 circRNAs (nine elevated and 10 suppressed) were recognized (Figures 1A,B). Due to the failure in synthesizing primers of some identified circRNA, the left eight elevated (hsa-circ-0072697, hsa-circ-0005286, hsa-circ-0003288, hsa-circ-0103279, hsa-circ-0005939, hsa-circ-0006427, hsa-circ-0006222, and hsa-circ-0088213) and 10 suppressed circRNAs (hsa-circ-0005078, hsa-circ-0084748, hsa-circ-0007440, hsa-circ-0035897, hsa-circ-0005204, hsa-circ-0137008, hsa-circ-0005238, hsa-circ-0086190, hsa-circ-0002590, hsa-circ-0000972) in FGR group are shown in Table 3.

**TABLE 3:** Statistically significant and differentially expressed circular RNAs (circRNAs).

Up-regulation circRNAs		Down-regulation circRNAs	
CircRNA	LogFC	CircRNA	LogFC
Hsa-circ-0072697	6.26	Hsa-circ-0005078	–5.76
Hsa-circ-0005286	5.34	Hsa-circ-0084748	–5.57
Hsa-circ-0003288	5.38	Hsa-circ-0007440	–5.42
Hsa-circ-0103279	5.18	Hsa-circ-0035897	–5.28
Hsa-circ-0005939	5.10	Hsa-circ-0005204	–5.56
Hsa-circ-0006427	5.06	Hsa-circ-0137008	–5.11
Hsa-circ-0006222	5.10	Hsa-circ-0005238	–5.07
Hsa-circ-0088213	2.54	Hsa-circ-0086190	–5.29
		Hsa-circ-0002590	–4.98
		Hsa-circ-0000972	–5.31

### GO and KEGG enrichment of differentially expressed circular RNAs

**FIGURE 2:**
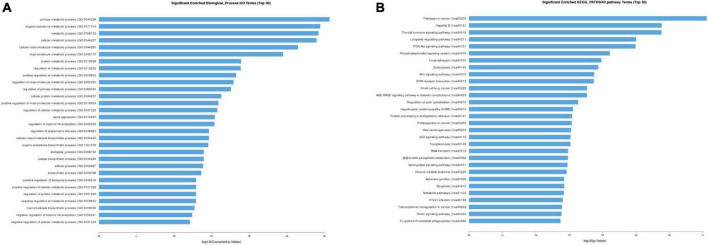
Gene ontology (GO) and kyoto encyclopedia of genes and genomes (KEGG) enrichment analysis. **(A)** Top 30 classes of biological process enrichment terms. **(B)** Top 30 KEGG pathway terms.

We further conducted gene ontology (GO) and kyoto encyclopedia of genes and genomes (KEGG) enrichment analyses to establish the biological processes (BPs), which these 19 differentially expressed circRNAs in FGR could act on. The top 10 markedly enriched BPs by GO enrichment were primary metabolic processes, organic substance metabolic processes, metabolic processes, cellular metabolic processes, cellular macromolecule metabolic processes, macromolecule metabolic process, protein metabolic process, regulation of metabolic process, positive regulation of metabolic process, and regulation of macromolecule metabolic process (Figure 2A). The top 10 enriched by KEGG were pathways in cancer, Hepatitis B, thyroid hormone signaling, longevity regulation, PI3K-Akt signaling, phosphatidyl inositol signaling system, focal adhesion, endocytosis, Wnt signaling pathway, ECM-receptor interaction (Figure 2B). In addition, we conducted GO enrichment analyses of hsa-circ-0005238 and found the top 10 enriched BPs were Golgi vesicle transport, tube morphogenesis, tissue morphogenesis, regulation of cell adhesion, cell leading edge, protein phosphorylation, gland development, cell adhesion molecule binding, cellular response to external stimulus, ribonucleoprotein granule (Supplementary Figure 3). We also found PI3K-Akt signaling pathway was ranked 13th in GO enrichment analyses. These results provided clues for further study on the regulation mechanisms of circRNA in FGR.

### Validation of the circular RNAs expression levels in fetal growth restriction tissue

**FIGURE 3:**
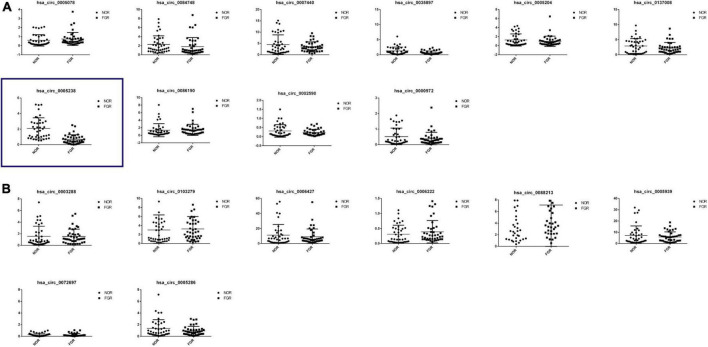
Reverse transcription quantitative PCR (qRT-PCR) confirmation of 18 differentially expressed circRNAs. **(A)** Expression profiles of 10 down-regulated differentially expressed circRNAs by RT-PCR were consistent with the NGS results. Hsa-circ-0005238 was displayed in the blue box, which was significantly downregulation in FGR group. **(B)** Expression profiles of nine up-rerulated differentially expressed circRNAs by RT-PCR were consistent with the NGS results.

Placental samples from 40 FGR women and 40 healthy pregnant women were obtained for validation of the differentiated expression of circRNAs. Relative levels of the 18 identified circRNAs were consistent with NGS data (Figures 3A,B). Then we checked for the specificity of qRT-PCR products by melting curve (Supplementary Figure 1A). Among the 18 circRNAs, hsa-circ-0005238, located at chr21:17205666-17214859 was the most influenced one and Student *t*-test showed most significant difference (*P* < 0.05), while other 17 circRNAs showed less significant statistical difference. The position of the splice junctions was verified by sequencing analyses of hsa-circ-0005238 fragment *via* qRT-PCR (Supplementary Figure 1B).

### Hsa-circ-0005238 inhibited apoptosis and enhanced proliferation, migration, invasion in trophoblast cells

**FIGURE 4:**
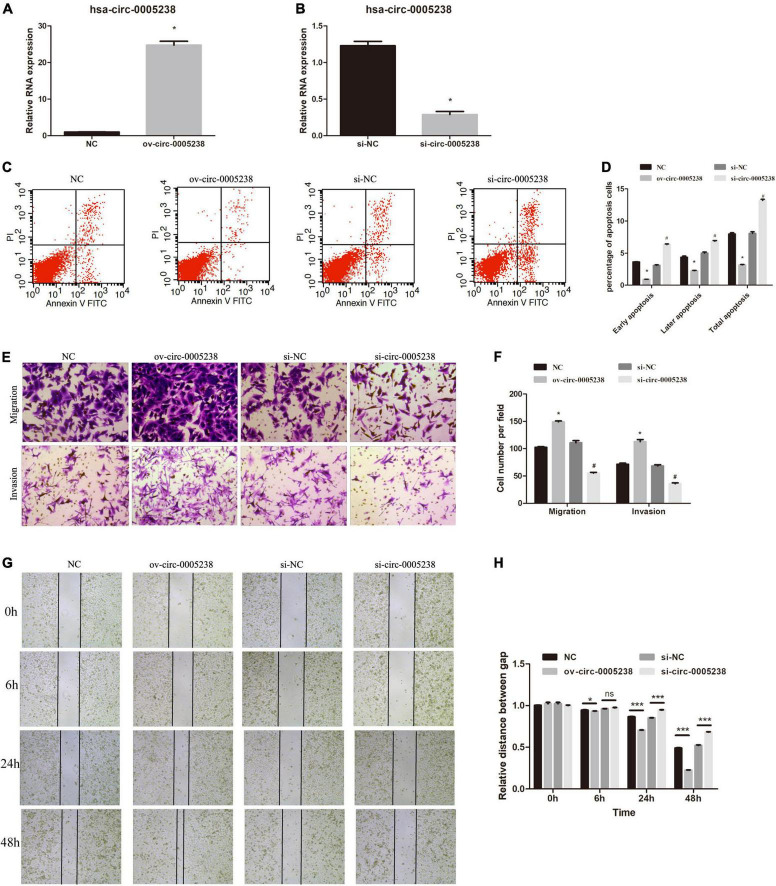
Effect of hsa-circ-0005238 overexpression or knockdown on biological functions of HTR-8 cells. **(A)** Hsa-circ-0005238 is overexpressed in HTR-8 cells after transfection ov-circ-0005238 compared to NC. **(B)** Hsa-circ-0005238 expression is knockdowned in HTR-8 cells after transfection with siRNA compared to si-NC. **(C)** Representative graphs of the apoptosis. **(D)** Quantification of the percentage of apoptotic cells. **(E)** Representative images of migration and invasion by Transwell assay and Boyden assay. **(F)** The quantification of migrated or invaded cell numbers. **(G)** Representative images of wound healing assay. **(H)** The relative distance between gap. ^#^*P* < 0.05, *P < 0.05, ***P < 0.001.

We further evaluated the function of hsa-circ-0005238 by silencing or overexpressing hsa-circ-0005238 in trophoblasts. After transfection with ov-circ-0005238, hsa-circ-0005238 levels were increased in HTR-8 cells compared with NC group (Figure 4A). Simultaneously, after transfection with si-circ-0005238, hsa-circ-0005238 levels were decreased in HTR-8 cells compared to si-NC group (Figure 4B).

The significance of hsa-circ-0005238 in apoptosis was detected by flow cytometry. As shown in Figures 4C,D, overexpression of hsa-circ-0005238 inhibited both early and late apoptosis in HTR-8 cells, while silence of hsa-circ-0005238 promoted both early and late apoptosis compared with the control.

As expected, overexpression of hsa-circ-0005238 enhanced cell proliferation (Supplementary Figure 1C), while silence of hsa-circ-0005238 suppressed cell proliferation (Supplementary Figure 1C) as compared with those in the negative controls.

Furthermore, the significance of hsa-circ-0005238 in cell migration was studied using Transwell assay. As shown in Figures 4E,F, ov-circ-0005238 group had more cells migrated to the bottom chamber, but the si-circ-0005238 group, by contrast, had less cells than the si-NC group. Besides, more cells invaded through the Matrigel in ov-circ-0005238 group, relative to NC group and less in si-circ-0005238 group, relative to si-NC group. In addition, the wound healing assay demonstrated that overexpression of hsa-circ-0005238 enhanced the migration capacity but the hsa-circ-0005238 knockdown inhibited the migration capacity (Figures 4G,H).

### Hsa-circ-0005238 targeted hsa-miR-370-3p

**FIGURE 5:**
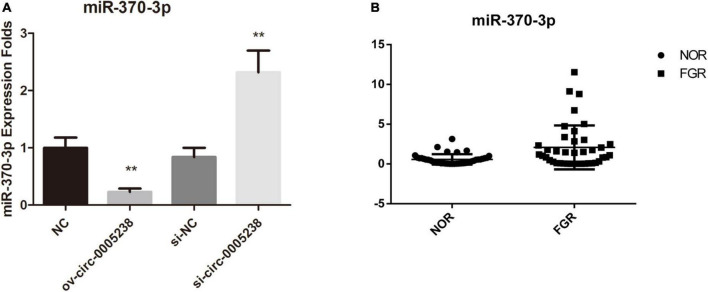
Expression of hsa-miR-370-3p in the ov-circ-0005238, si-circ-0005238 group, and FGR placenta. **(A)** Expression of miR-370-3p in ov-circ-0005238, ov-NC, si-circ-0005238, and si-NC group. **(B)** hsa-miR-370-3p expression in FGR or normal placental tissues (*n* = 40). **P < 0.01.

Follow online bioinformatics prediction by TargetScan, we screened four microRNA which had high context + score percentile as candidate microRNA–miR-6893-3p, miR-370-3p, miR-3065-5p, miR-5585-3p. We first investigated the expression of these four microRNAs in ov-circ-0005238, ov-NC, si-circ-0005238, and si-NC HTR-8 cells. The hsa-miR-370-3p levels were markedly depressed in ov-circ-0005238 group relative to ov-NC group (*P* = 0.002), while its expression was risen in the si- circ-0005238 group relative to si-NC group (*P* = 0.003) (Figure 5A), implying that hsa-circ-0005238 might be a miR-370-3p sponge. We verified hsa-miR-370-3p levels in 40 FGR and 40 normal placenta tissues. Hsa-miR-370-3p levels were markedly elevated in FGR placenta tissues (Figure 5B).

**FIGURE 6:**
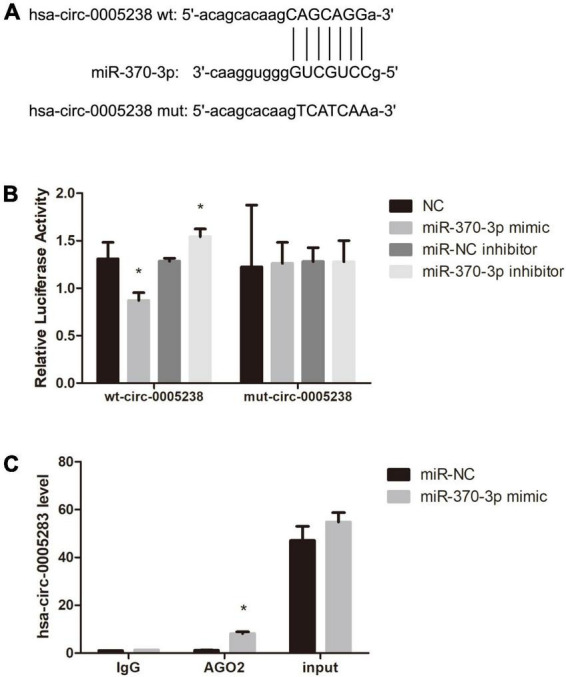
Hsa-miR-370-3p was targeted by hsa-circ-0005238 in HTR-8 cells. **(A)** The binding site of hsa-miR-370-3p on hsa-circ-0005238. **(B)** Results of the dual luciferase activity assay. **(C)** Results of the anti-AGO2 immunoprecipitation (RIP) assay. *P < 0.05.

Moreover, dual luciferase activity analysis was to confirm the binding of hsa-miR-370-3p to linear hsa-circ-0005238. We predicted the binding site of hsa-miR-370-3p on wild type hsa-circ-0005238 (Figure 6A). Figure 6A also showed the sequence of the mutant hsa-circ-0005238. Figure 6B showed findings from dual luciferase activity assay. After hsa-miR-370-3p mimic + wt-circ-0005238 transfection, relative luciferase activities were lower than after NC transfection, while relative luciferase activities were high after hsa-miR-370-3p inhibitor + wt-circ-0005238 transfections. However, the mut-circ-0005238 transfection group failed to get the same results. On the other hand, anti-AGO2 RIP analysis was performed to confirm if hsa-miR-370-3p binds hsa-circ-0005238. As shown in Figure 6C, hsa-circ-0005238 levels in RIP product of miR-370-3p -transfected cells were high relative to those in miR-NC-transfected cells. To summarize what had been mentioned above, hsa-miR-370-3p bound the endogenous hsa-circ-0005238 in HTR-8 cells.

### Hsa-miR-370-3p overexpression reversed the effects of overexpressed hsa-circ-0005238 HTR-8 cells

**FIGURE 7:**
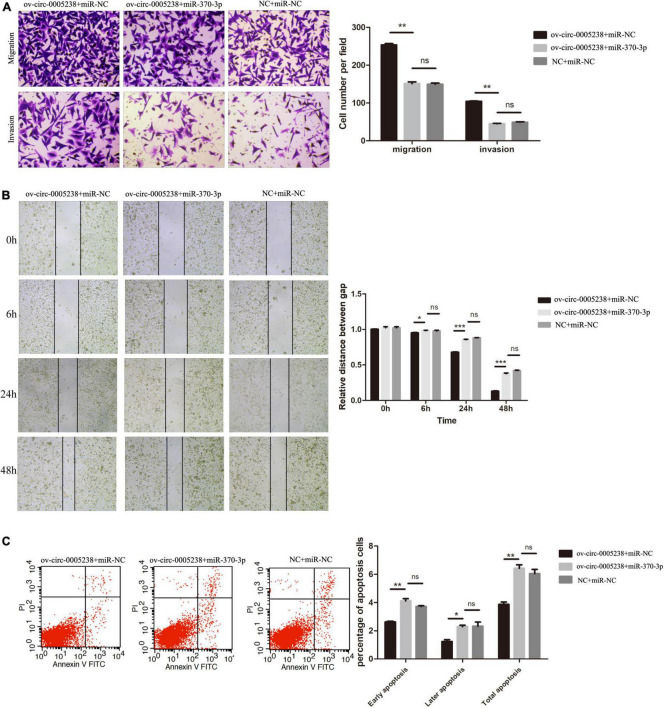
Hsa-miR-370-3p overexpression eliminated the effect of hsa-circ-0005238 overexpression in HTR-8 cells. **(A)** Representative images and quantification of migration and invasion. **(B)** Representative images of wound healing assay and the relative distance between gap. **(C)** Representative images and quantification of apoptosis. *P < 0.05, **P < 0.01, ***P < 0.001.

To verify the above findings, hsa-miR-370-3p mimics and hsa-circ-0005238 overexpressing plasmid were transfected at the same time into HTR-8 cells. Cell proliferation was suppressed in ov-circ-0005238 + miR-370-3p group, as compared with ov-circ-0005238 + miR-NC group (Supplementary Figure 1D). Figure 7A shows that cell number of the ov-circ-0005238 + miR-370-3p group migrating to bottom chamber was lower than that in ov-circ-0005238 + miR-NC group. Meanwhile, cells invasion showed the similar results as migration. Besides, Figure 7B also shows that cell migration was reduced in ov-circ-0005238 + miR-370-3p group compared with the ov-circ-0005238 + miR-NC group. In ov-circ-0005238 + miR-370-3p group, on the other hand, the proportion of apoptotic cells was high relative to ov-circ-0005238 + miR-NC group as shown in Figure 7C. Furthermore, there were no significantly differences between NC + miR-NC and ov-circ-0005238 + miR-370-3p groups in the migration, invasion or apoptotic assays. Thus, hsa-miR-370-3p overexpression reversed hsa-circ-0005238 overexpression-mediated HTR-8 cell-migration, invasion as well as apoptosis.

### Hsa-miR-370-3p inhibitor downregulated apoptosis and promoted HTR-8 cell migration and invasion

**FIGURE 8:**
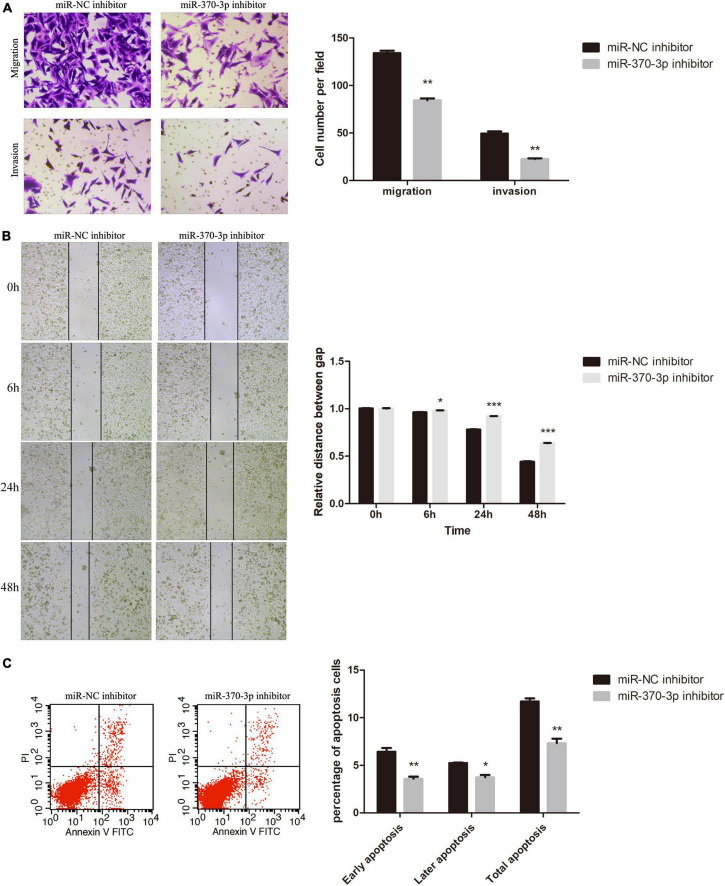
Effect of hsa-miR-370-3p knockdown on apoptosis, migration and invasion of HTR-8 cells. **(A)** Representative images and quantification of migration and invasion. **(B)** Representative images of wound healing assay and the relative distance between gap. **(C)** Representative images and quantification of apoptosis. *P < 0.05, **P < 0.01, ***P < 0.001.

Next, we evaluated the significance of hsa-miR-370-3p in FGR. Transwell assays (Figure 8A) and the wound healing (Figure 8B) demonstrated that migration capacity of miR-370-3p inhibitor group was inhibited compared with that in the miR-NC inhibitor group. Cells invasion (Figure 8A) showed the resemble results as migration. Finally, the proportion of apoptotic cells in miR-370-3p inhibitor group was low than that of miR-NC inhibitor group (Figure 8C).

### CDC25B is a downstream target of hsa-miR-370-3p

**FIGURE 9:**
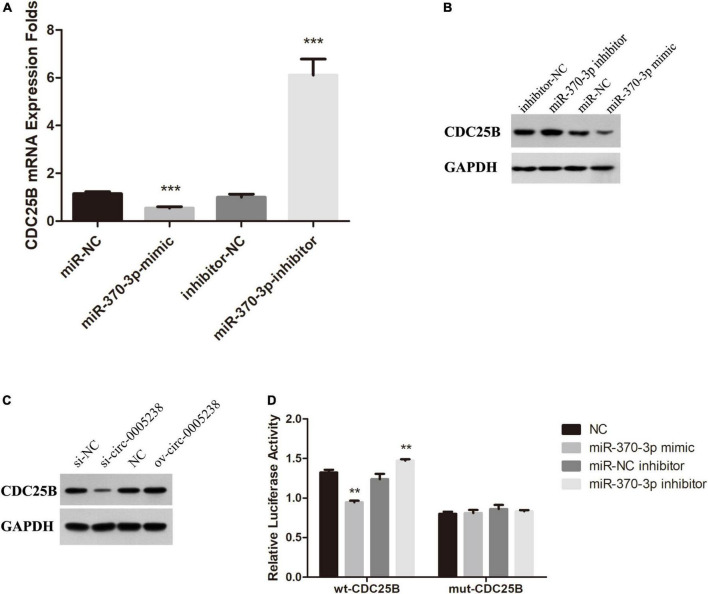
CDC25B is a direct target of miR-370-3p. **(A)** Expression of CDC25B in miR-370-3p-mimic, miR-NC, inhibitor- miR-370-3p and inhibitor-NC group by RT-qPCR. **(B)** Western Blot results of CDC25B expression in miR-370-3p-mimic, miR-NC, inhibitor- miR-370-3p and inhibitor-NC group. **(C)** Western Blot results of CDC25B expression in si-circ-0005238, si-NC, ov-circ-0005238 and NC group. **(D)** Results of the dual luciferase activity assay. **P < 0.01, ***P < 0.001.

Subsequently, we aimed to explore the mechanisms of hsa-miR-370-3p so we predicted the target mRNAs by online bioinformatics prediction on miRDB^30^. Supplementary Table 1 shows that miRDB calculated with high context + score percentile and predicted 609 target mRNAs, among which five target mRNAs were candidate—GPRAB, PTPRB, CLOCK, FMR1, and CDC25B. Therefore, the five target mRNAs above were chosen to further identified. We first investigated the levels of these five target mRNAs in miR-370-3p-mimic, miR-NC, miR-370-3p-inhibitor, and inhibitor-NC HTR-8 cells using qRT-PCR and western blots (Supplementary Figure 2). We found that CDC25B levels were significantly low in miR-370-3p-mimic group, relative to miR-NC group, while its expression was high in miR-370-3p-inhibitor group relative to inhibitor-NC group (*P* < 0.001) (Figures 9A,B). In addition, CDC25B levels were reduced after hsa-circ-0005238 knockdown but CDC25B levels were increased after hsa-circ-0005238 overexpression. These results above indicates that CDC25B is the target mRNA of miR-370-3p (Figure 9C).

Figure 9D shows that relative luciferase activities were low after hsa-miR-370-3p mimic transfection, relative to NC transfection, while the relative luciferase activities were high after hsa-miR-370-3p inhibitor transfections, relative to miR-NC inhibitor transfection. However, the mut-CDC25B transfection group was unable to come to the same results. It indicated that hsa-miR-370-3p mimic bound the sequence of CDC25B cloned in psi-CHECK2. These findings imply that CDC25B acted as a downstream target for miR-370-3p in HTR-8 cells.

## Discussion

Fetal growth restriction is described as a failure of the fetus to reach its growth and developmental potential. Etiology of FGR is multifactorial, such as maternal causes, fetal causes, and causes involving placental insufficiency. Currently the incidence rates of FGR is the highest over the last decades and it is likely to grow, which becomes a global public health challenge (20). As a newly discovered non-coding RNA, researchers all over the world pay great attention on circRNA. Nowadays, there are various methods which can be used to examine circRNA profiling, such as RT-qPCR, microarrays, and NGS. Each method has its advantages and limitations; however, NGS have better sensitivity and capability of generating quantification. Neoteric bioinformatic strategies combined with biochemical enrichment approaches have allowed people to study circRNAs comprehensively. Circular RNAs play an important role in human diseases (21). Many are predictive biomarkers and have the potential to be therapeutic targets for therapy (22, 23). Up to now, the association between FGR and circRNAs has not been clearly established.

Maass et al. (24) documented that several circRNAs of placenta are involved in pregnancy complications, including fetal growth restriction, pre-eclampsia, HELLP syndrome, and diabetes. Bai et al. (25) found some circRNAs, which were differentially expressed in the placenta tissue, contributed to the pathogenesis of preeclampsia. A placental villi circRNA screening from the Tang et al. (26) found 55 upregulated circRNAs and 59 suppressed circRNAs between gestational diabetes mellitus (GDM) patients and normal pregnancies. CircRNAs are potential miRNA sponges to regulate genes expression. Wang et al. (27) provided hsa-circ-0000848 modulate the trophoblast cell function *via* the sponging of hsa-miR-370-3p. Nowadays, most studies of FGR focused on maternal causes. However, those placenta samples with maternal or fetal conditions leading to FGR were excluded from the study. According to our study, circRNAs may act as suitable biomarkers for uterine–placental perfusion, placental maldevelopment and insufficiency.

In this study, after all maternal and fetal causes were excluded from our samples, we characterized placenta-specific circRNAs in FGR. Firstly, by NGS, 18 placenta-specific circRNAs (hsa-circ-0005078, hsa-circ-0072697, hsa-circ-0084748, hsa-circ-0007440, hsa-circ-0005286, hsa-circ-0003288, hsa-circ-0035897, hsa-circ-0103279, hsa-circ-0005204, hsa-circ-0005939, hsa-circ-0006427, hsa-circ-0137008, hsa-circ-0006222, hsa-circ-0005238, hsa-circ-0088213, hsa-circ-0086190, hsa-circ-0002590, hsa-circ-0000972) were found. Thus far, hsa-circ-0137008 was reported to suppress the malignant phenotype in colorectal cancer cells (28). Hsa-circ-0003288 facilitates malignant phenotype in hepatocellular carcinoma and non-small cell lung cancer (29, 30). Other 16 circRNAs were reported for the first time.

We characterized 18 placenta-specific circRNAs in FGR by NGS. The potential function of the differentially expressed circRNAs in FGR were analyzed according to the GO and KEGG enrichment analyses. As mentioned above, we found these differentially expressed circRNAs were enriched in metabolic and morphogenesis biological processes or signaling pathways, which suggested that circRNA may take part in the early stage of FGR. Among differentially expressed circRNAs in FGR, hsa-circ-0005238 attracted our attention. Hsa-circ-0005238 was notably suppressed in placenta of FGR women, which has been not reported in pregnancy. We found that overexpressed hsa-circ-0005238 inhibited, while its knockdown enhanced apoptosis in trophoblast cells. Moreover, overexpressed hsa-circ-0005238 promoted, while its knockdown suppressed trophoblast cell proliferation, cell migration as well as invasion. During pregnancy, placental trophoblasts cells move upstream along arterial walls, replace endothelia, and dysregulate muscular linings (31), these kind of migration and invasion were in a manner not different from most aggressive tumors. Only then, the trophoblast invasion during human placentation enables fetus to derive nutrition from mater. In this study, KEGG pathways enrichment showed the top one pathway was cancer. There are so much similarities between trophoblast cells into the maternal uterus and the growth of cancer cells (32). In addition, the development of placental involves a complex but strict gene regulation (33). The placenta, therefore, act as a pseudomalignant tissue. Abnormal growth, migration, and invasion of trophoblast cells may result in abnormal pregnancy, such as FGR. Thus, we conjectured that hsa-circ-0005238 has important roles in FGR pathogenesis. Nevertheless, its mechanisms were needed to be confirm by further experiments.

With regards to regulatory models of circRNAs as microRNAs sponges, inhibitory effects of miRNAs on target genes is ameliorated, while target gene expressions are enhanced *via* a competitive endogenous RNA mechanism (34). First, we identified hsa-miR-370-3p as a potential target miRNA of hsa-circ-0005238. MiR-370-3p was revealed as a tumor promoter in breast cancer (35) and gastric cancer (36). miR-370-3p was participate in fetal adrenal developmental programming (37). However, how miR-370-3p are involved in FGR is unclear until now. Further, we found hsa-miR-370-3p was increased in the placentas of FGR women. In addition, hsa-circ-0005238 downregulation enhanced hsa-miR-370-3p levels in HTR-8 cells. Then hsa-circ-0005238 was confirmed as a sponge for hsa-miR-370-3p. MiRNAs suppress the translation of target mRNA or are involved in its degradation. In our work, hsa-miR-370-3p overexpression decreased CDC25B expression in HTR-8 cells and hsa-circ-0005238 overexpression increased CDC25B expression in HTR-8 cells. In addition, luciferase reporter proved CDC25B served as a target mRNA for miR-370-3p in trophoblasts. CDC25B is a member of phosphatases and key gene for entry into mitosis. Overexpressed CDC25B promotes the entry of cells into mitosis before the usual time and shows spindle abnormalities (38). Comparatively, depleted CDC25B delays mitotic entry (38). Lam et al. (39) reported a unique Chinese girl with retarded intrauterine growth as well as adolescent delayed development and she was diagnosed as homozygous non-sense variant in the CDC25B gene by whole-exome sequencing analysis. Several studies elegantly substantiated that CDC25B enhances cell migration and invasion (40, 41). It is suggested that CDC25B may be potentially therapeutic for FGR. Regrettably, we did not make a profound study the downstream pathway of hsa-circ-0005238/miR-370-3p/CDC25B axis.

In summary, we demonstrate placenta-specific circRNAs in FGR pregnancy. Our data indicates that the downgraded expression of FGR placenta-specific hsa-circ-0005238 may take part in the occurrence and pathogenesis of FGR *via* decreasing CDC25B expression through sponging miR-370-3p. Our study also elucidates on pathomechanisms of FGR.

## Data availability statement

The datasets presented in this study can be found in online repositories. The names of the repository/repositories and accession number(s) can be found below: https://db.cngb.org/cnsa/, CNP 0002703.

## Ethics statement

The studies involving human participants were reviewed and approved by Medical Ethics Committee of Shenzhen Maternity and Child Healthcare Hospital ([2018]280). The patients/participants provided their written informed consent to participate in this study.

## Author contributions

ZH, QZ, and JY conceived and designed the research and edited and revised the manuscript. ZH performed the experiments. ZH and LZ analyzed the data. ZH, LZ, and DZ prepared the figures and drafted the manuscript. All authors contributed to the article and approved the submitted version.

## Footnotes

1
http://www.circbase.org

2
http://www.targetscan.org/vert_71/

3
https://db.cngb.org/cnsa/

## Supplementary material

Supplementary Figure 1 **(A)** Supplementary melting curve analysis of hsa-circ-0005238. **(B)** Sequencing analysis around the splice junction of hsa-circ-0005238 fragment in RT-qPCR. **(C)** Cell proliferation results of hsa-circ-0005238 overexpression or knockdown. **(D)** Cell proliferation results of reverse experiment. **P* < 0.05, ^**^*P* < 0.01, ^***^*P* < 0.001.

Supplementary Figure 2 Expression of GPRAB, PTPRB, CLOCK, and FMR1 in miR-370-3p-mimic, miR-NC, inhibitor- miR-370-3p, and inhibitor-NC group by RT-qPCR. **(A)** Expression of GPRAB. **(B)** Expression of PTPRB. **(C)** Expression of CLOCK. **(D)** Expression of FMR1.

Supplementary Figure 3 Top 20 pathway terms in GO enrichment analysis of hsa-circ-0005238.
