# Supplementary material for: Circular RNA hsa-circ-0005238 enhances trophoblast migration, invasion and suppresses apoptosis via the miR-370-3p/CDC25B axis
Source: Front Med (Lausanne). 2022 Oct 13;9:943885. doi: 10.3389/fmed.2022.943885 (PMC9606333; doi:10.3389/fmed.2022.943885)
Supplement: Supplementary file 1 [file Data_Sheet_1.PDF]

Target Rank	Target Score	miRNA Name	Gene Symbol	Gene Description
1	97	hsa-miR-370-3p	INO80	INO80 complex subunit
2	97	hsa-miR-370-3p	ATXN7L3	ataxin 7 like 3
3	96	hsa-miR-370-3p	ATP11A	ATPase phospholipid transporting 11A
4	95	hsa-miR-370-3p	ZCCHC17	zinc finger CCHC-type containing 17
5	95	hsa-miR-370-3p	AR	androgen receptor
6	94	hsa-miR-370-3p	SMURF1	SMAD specific E3 ubiquitin protein ligase 1
7	94	hsa-miR-370-3p	MAU2	MAU2 sister chromatid cohesion factor
8	93	hsa-miR-370-3p	CHD2	chromodomain helicase DNA binding protein 2
9	93	hsa-miR-370-3p	ING3	inhibitor of growth family member 3
10	93	hsa-miR-370-3p	DCBLD1	discoidin, CUB and LCCL domain containing 1
11	92	hsa-miR-370-3p	SPTSSB	serine palmitoyltransferase small subunit B
12	92	hsa-miR-370-3p	HMGA2	high mobility group AT-hook 2
13	92	hsa-miR-370-3p	CNTNAP1	contactin associated protein 1
14	92	hsa-miR-370-3p	MLEC	malectin
15	92	hsa-miR-370-3p	CHST11	carbohydrate sulfotransferase 11
16	92	hsa-miR-370-3p	KLF12	Kruppel like factor 12
17	91	hsa-miR-370-3p	TADA1	transcriptional adaptor 1
18	91	hsa-miR-370-3p	FLT1	fms related tyrosine kinase 1
19	91	hsa-miR-370-3p	RAP1GDS1	Rap1 GTPase-GDP dissociation stimulator 1
20	91	hsa-miR-370-3p	SEZ6	seizure related 6 homolog
21	91	hsa-miR-370-3p	VPS26A	VPS26, retromer complex component A
22	90	hsa-miR-370-3p	SRSF6	serine and arginine rich splicing factor 6
23	90	hsa-miR-370-3p	RFX7	regulatory factor X7
24	90	hsa-miR-370-3p	PRTFDC1	phosphoribosyl transferase domain containing 1
25	90	hsa-miR-370-3p	RNF20	ring finger protein 20
26	89	hsa-miR-370-3p	SLC4A4	solute carrier family 4 member 4
27	89	hsa-miR-370-3p	MBNL1	muscleblind like splicing regulator 1
28	88	hsa-miR-370-3p	SCG3	secretogranin III
29	88	hsa-miR-370-3p	LRRK1	leucine rich repeat kinase 1
30	88	hsa-miR-370-3p	OSCAR	osteoclast associated, immunoglobulin-like receptor
31	88	hsa-miR-370-3p	USP37	ubiquitin specific peptidase 37
32	87	hsa-miR-370-3p	CHST15	carbohydrate sulfotransferase 15
33	87	hsa-miR-370-3p	WNT10B	Wnt family member 10B
34	87	hsa-miR-370-3p	PLEKHG4B	pleckstrin homology and RhoGEF domain containing G4B
35	86	hsa-miR-370-3p	SMAD1	SMAD family member 1
36	86	hsa-miR-370-3p	GSS	glutathione synthetase
37	86	hsa-miR-370-3p	SEC22C	SEC22 homolog C, vesicle trafficking protein
38	86	hsa-miR-370-3p	TRIM33	tripartite motif containing 33
39	86	hsa-miR-370-3p	ADGRA2	adhesion G protein-coupled receptor A2
40	86	hsa-miR-370-3p	CDC42EP4	CDC42 effector protein 4
41	86	hsa-miR-370-3p	CES3	carboxylesterase 3
42	86	hsa-miR-370-3p	PMAIP1	phorbol-12-myristate-13-acetate-induced protein 1
43	86	hsa-miR-370-3p	GPD1	glycerol-3-phosphate dehydrogenase 1
44	85	hsa-miR-370-3p	DOCK4	dedicator of cytokinesis 4
45	85	hsa-miR-370-3p	NR4A3	nuclear receptor subfamily 4 group A member 3
46	85	hsa-miR-370-3p	DBX2	developing brain homeobox 2
47	84	hsa-miR-370-3p	FOXO1	forkhead box O1
48	84	hsa-miR-370-3p	ZDHHC5	zinc finger DHHC-type containing 5
49	84	hsa-miR-370-3p	FAM193A	family with sequence similarity 193 member A
50	84	hsa-miR-370-3p	DNMT3B	DNA methyltransferase 3 beta
51	84	hsa-miR-370-3p	SERTAD4	SERTA domain containing 4
52	84	hsa-miR-370-3p	TMEM161B	transmembrane protein 161B
53	84	hsa-miR-370-3p	TPCN1	two pore segment channel 1
54	84	hsa-miR-370-3p	CEP112	centrosomal protein 112

55	84	hsa-miR-370-3p	ARMC7	armadillo repeat containing 7
56	84	hsa-miR-370-3p	RAPGEF1	Rap guanine nucleotide exchange factor 1
57	84	hsa-miR-370-3p	DHX35	DEAH-box helicase 35
58	84	hsa-miR-370-3p	APOBEC3F	apolipoprotein B mRNA editing enzyme catalytic subunit 3F
59	84	hsa-miR-370-3p	EXOC7	exocyst complex component 7
60	83	hsa-miR-370-3p	GRIP1	glutamate receptor interacting protein 1
61	83	hsa-miR-370-3p	COX16	cytochrome c oxidase assembly factor COX16
62	83	hsa-miR-370-3p	BICD2	BICD cargo adaptor 2
63	83	hsa-miR-370-3p	UPF1	UPF1, RNA helicase and ATPase
64	83	hsa-miR-370-3p	SYNJ2BP-COX16	SYNJ2BP-COX16 readthrough
65	83	hsa-miR-370-3p	ADCY1	adenylate cyclase 1
66	83	hsa-miR-370-3p	MGMT	O-6-methylguanine-DNA methyltransferase
67	83	hsa-miR-370-3p	BAZ2A	bromodomain adjacent to zinc finger domain 2A
68	83	hsa-miR-370-3p	IFIT1	interferon induced protein with tetratricopeptide repeats 1
69	83	hsa-miR-370-3p	ZNF189	zinc finger protein 189
70	83	hsa-miR-370-3p	HNRNPL	heterogeneous nuclear ribonucleoprotein L
71	82	hsa-miR-370-3p	PSD3	pleckstrin and Sec7 domain containing 3
72	82	hsa-miR-370-3p	CASKIN1	CASK interacting protein 1
73	82	hsa-miR-370-3p	ATXN1	ataxin 1
74	82	hsa-miR-370-3p	PEAK1	pseudopodium enriched atypical kinase 1
75	82	hsa-miR-370-3p	SMAP1	small ArfGAP 1
76	82	hsa-miR-370-3p	DSTYK	dual serine/threonine and tyrosine protein kinase
77	82	hsa-miR-370-3p	TPM4	tropomyosin 4
78	82	hsa-miR-370-3p	ZNF827	zinc finger protein 827
79	81	hsa-miR-370-3p	SLAMF6	SLAM family member 6
80	81	hsa-miR-370-3p	MTMR1	myotubularin related protein 1
81	81	hsa-miR-370-3p	ZDHHC15	zinc finger DHHC-type containing 15
82	81	hsa-miR-370-3p	NFASC	neurofascin
83	81	hsa-miR-370-3p	TPO	thyroid peroxidase
84	81	hsa-miR-370-3p	CCDC198	coiled-coil domain containing 198
85	81	hsa-miR-370-3p	CD84	CD84 molecule
86	81	hsa-miR-370-3p	TMEM117	transmembrane protein 117
87	81	hsa-miR-370-3p	AP3M1	adaptor related protein complex 3 subunit mu 1
88	80	hsa-miR-370-3p	PAG1	phosphoprotein membrane anchor with glycosphingolipid microdomains 1
89	80	hsa-miR-370-3p	USP39	ubiquitin specific peptidase 39
90	80	hsa-miR-370-3p	SOGA1	suppressor of glucose, autophagy associated 1
91	80	hsa-miR-370-3p	ERVV-2	endogenous retrovirus group V member 2, envelope
92	80	hsa-miR-370-3p	AFF1	AF4/FMR2 family member 1
93	80	hsa-miR-370-3p	NDST1	N-deacetylase and N-sulfotransferase 1
94	80	hsa-miR-370-3p	OR12D3	olfactory receptor family 12 subfamily D member 3
95	80	hsa-miR-370-3p	SH3PXD2A	SH3 and PX domains 2A
96	80	hsa-miR-370-3p	PIK3CA	phosphatidylinositol-4,5-bisphosphate 3-kinase catalytic subunit alpha
97	80	hsa-miR-370-3p	MAP4K4	mitogen-activated protein kinase kinase kinase kinase 4
98	80	hsa-miR-370-3p	ZBTB37	zinc finger and BTB domain containing 37
99	80	hsa-miR-370-3p	TMEM132B	transmembrane protein 132B
100	80	hsa-miR-370-3p	BAG4	BCL2 associated athanogene 4
101	79	hsa-miR-370-3p	TMEM266	transmembrane protein 266
102	79	hsa-miR-370-3p	ITGB1BP2	integrin subunit beta 1 binding protein 2
103	79	hsa-miR-370-3p	ENOSF1	enolase superfamily member 1
104	79	hsa-miR-370-3p	NF1	neurofibromin 1
105	79	hsa-miR-370-3p	SPDYE1	speedy/RINGO cell cycle regulator family member E1
106	79	hsa-miR-370-3p	SPDYE3	speedy/RINGO cell cycle regulator family member E3

107	79	hsa-miR-370-3p	BACH2	BTB domain and CNC homolog 2
108	79	hsa-miR-370-3p	ANK3	ankyrin 3
109	79	hsa-miR-370-3p	SPDYE6	speedy/RINGO cell cycle regulator family member E6
110	79	hsa-miR-370-3p	CCNJ	cyclin J
111	79	hsa-miR-370-3p	GLYATL1	glycine-N-acyltransferase like 1
112	79	hsa-miR-370-3p	SPDYE5	speedy/RINGO cell cycle regulator family member E5
113	79	hsa-miR-370-3p	DDHD2	DDHD domain containing 2
114	79	hsa-miR-370-3p	CCDC141	coiled-coil domain containing 141
115	79	hsa-miR-370-3p	KLHL18	kelch like family member 18
116	78	hsa-miR-370-3p	KLHDC7B	kelch domain containing 7B
117	78	hsa-miR-370-3p	TREM1	triggering receptor expressed on myeloid cells 1
118	78	hsa-miR-370-3p	MYF6	myogenic factor 6
119	78	hsa-miR-370-3p	XPO4	exportin 4
120	78	hsa-miR-370-3p	ABCG4	ATP binding cassette subfamily G member 4
121	78	hsa-miR-370-3p	URB1	URB1 ribosome biogenesis homolog
122	78	hsa-miR-370-3p	TMEM154	transmembrane protein 154
123	78	hsa-miR-370-3p	TFDP2	transcription factor Dp-2
124	78	hsa-miR-370-3p	PARVB	parvin beta
125	78	hsa-miR-370-3p	COMMD7	COMM domain containing 7
126	77	hsa-miR-370-3p	RGS6	regulator of G protein signaling 6
127	77	hsa-miR-370-3p	BEX2	brain expressed X-linked 2
128	77	hsa-miR-370-3p	ZC3H7B	zinc finger CCCH-type containing 7B
129	77	hsa-miR-370-3p	CCDC25	coiled-coil domain containing 25
130	77	hsa-miR-370-3p	FAM219B	family with sequence similarity 219 member B
131	77	hsa-miR-370-3p	VAV2	vav guanine nucleotide exchange factor 2
132	77	hsa-miR-370-3p	CIT	citron rho-interacting serine/threonine kinase
133	77	hsa-miR-370-3p	TM9SF4	transmembrane 9 superfamily member 4
134	76	hsa-miR-370-3p	IL18BP	interleukin 18 binding protein
135	76	hsa-miR-370-3p	CASTOR2	cytosolic arginine sensor for mTORC1 subunit 2
136	76	hsa-miR-370-3p	HSPA12A	heat shock protein family A (Hsp70) member 12A
137	76	hsa-miR-370-3p	CASK	calcium/calmodulin dependent serine protein kinase
138	76	hsa-miR-370-3p	EML1	EMAP like 1
139	76	hsa-miR-370-3p	FCGR3A	Fc fragment of IgG receptor IIIa
140	76	hsa-miR-370-3p	FCGR3B	Fc fragment of IgG receptor IIIb
141	75	hsa-miR-370-3p	MGLL	monoglyceride lipase
142	75	hsa-miR-370-3p	RCBTB1	RCC1 and BTB domain containing protein 1
143	75	hsa-miR-370-3p	CASQ2	calsequestrin 2
144	75	hsa-miR-370-3p	HERPUD1	homocysteine inducible ER protein with ubiquitin like domain 1
145	75	hsa-miR-370-3p	LAYN	layilin
146	75	hsa-miR-370-3p	SOX12	SRY-box 12
147	75	hsa-miR-370-3p	NKAIN1	sodium/potassium transporting ATPase interacting 1
148	75	hsa-miR-370-3p	VDAC2	voltage dependent anion channel 2
149	75	hsa-miR-370-3p	CD40	CD40 molecule
150	75	hsa-miR-370-3p	TNRC6B	trinucleotide repeat containing 6B
151	74	hsa-miR-370-3p	ACP7	acid phosphatase 7, tartrate resistant (putative)
152	74	hsa-miR-370-3p	ASCC1	activating signal cointegrator 1 complex subunit 1
153	74	hsa-miR-370-3p	ITM2C	integral membrane protein 2C
154	74	hsa-miR-370-3p	SPATA2L	spermatogenesis associated 2 like
155	74	hsa-miR-370-3p	UPF2	UPF2, regulator of nonsense mediated mRNA decay
156	74	hsa-miR-370-3p	LDLRAP1	low density lipoprotein receptor adaptor protein 1
157	74	hsa-miR-370-3p	SLC24A4	solute carrier family 24 member 4
158	74	hsa-miR-370-3p	TRIB2	tribbles pseudokinase 2
159	74	hsa-miR-370-3p	DUSP3	dual specificity phosphatase 3
160	74	hsa-miR-370-3p	TRAIP	TRAF interacting protein
161	74	hsa-miR-370-3p	COL26A1	collagen type XXVI alpha 1 chain

162	73	hsa-miR-370-3p	SV2C	synaptic vesicle glycoprotein 2C
163	73	hsa-miR-370-3p	EPHB2	EPH receptor B2
164	73	hsa-miR-370-3p	PDPR	pyruvate dehydrogenase phosphatase regulatory subunit
165	73	hsa-miR-370-3p	KLHL6	kelch like family member 6
166	73	hsa-miR-370-3p	PAX5	paired box 5
167	73	hsa-miR-370-3p	NCOA5	nuclear receptor coactivator 5
168	73	hsa-miR-370-3p	POMT2	protein O-mannosyltransferase 2
169	73	hsa-miR-370-3p	PRND	prion like protein doppel
170	73	hsa-miR-370-3p	FRK	fyn related Src family tyrosine kinase
171	73	hsa-miR-370-3p	BAP1	BRCA1 associated protein 1
172	73	hsa-miR-370-3p	ZNF397	zinc finger protein 397
173	72	hsa-miR-370-3p	GPR62	G protein-coupled receptor 62
174	72	hsa-miR-370-3p	CCDC170	coiled-coil domain containing 170
175	72	hsa-miR-370-3p	ASIC4	acid sensing ion channel subunit family member 4
176	72	hsa-miR-370-3p	HDAC4	histone deacetylase 4
177	72	hsa-miR-370-3p	TFAM	transcription factor A, mitochondrial
178	72	hsa-miR-370-3p	DHX33	DEAH-box helicase 33
179	72	hsa-miR-370-3p	SNAP23	synaptosome associated protein 23
180	72	hsa-miR-370-3p	PRKACB	protein kinase cAMP-activated catalytic subunit beta
181	72	hsa-miR-370-3p	AJAP1	adherens junctions associated protein 1
182	72	hsa-miR-370-3p	STK35	serine/threonine kinase 35
183	72	hsa-miR-370-3p	MTMR3	myotubularin related protein 3
184	72	hsa-miR-370-3p	STMN4	stathmin 4
185	72	hsa-miR-370-3p	CNPY1	canopy FGF signaling regulator 1
186	71	hsa-miR-370-3p	RAPGEFL1	Rap guanine nucleotide exchange factor like 1
187	71	hsa-miR-370-3p	FBLN5	fibulin 5
188	71	hsa-miR-370-3p	CLEC2A	C-type lectin domain family 2 member A
189	71	hsa-miR-370-3p	ZNF248	zinc finger protein 248
190	71	hsa-miR-370-3p	STAT3	signal transducer and activator of transcription 3
191	71	hsa-miR-370-3p	DENND5A	DENN domain containing 5A
192	71	hsa-miR-370-3p	TMTC1	transmembrane and tetratricopeptide repeat containing 1
193	71	hsa-miR-370-3p	EPHA5	EPH receptor A5
194	71	hsa-miR-370-3p	RRP1B	ribosomal RNA processing 1B
195	71	hsa-miR-370-3p	SZT2	SZT2, KICSTOR complex subunit
196	71	hsa-miR-370-3p	PDE4DIP	phosphodiesterase 4D interacting protein
197	71	hsa-miR-370-3p	KAT6A	lysine acetyltransferase 6A
198	71	hsa-miR-370-3p	STK16	serine/threonine kinase 16
199	71	hsa-miR-370-3p	FGF7	fibroblast growth factor 7
200	71	hsa-miR-370-3p	C3orf62	chromosome 3 open reading frame 62
201	70	hsa-miR-370-3p	UVRAG	UV radiation resistance associated
202	70	hsa-miR-370-3p	MKNK1	MAP kinase interacting serine/threonine kinase 1
203	70	hsa-miR-370-3p	CYB561D1	cytochrome b561 family member D1
204	70	hsa-miR-370-3p	UFM1	ubiquitin fold modifier 1
205	70	hsa-miR-370-3p	TENM2	teneurin transmembrane protein 2
206	70	hsa-miR-370-3p	TMEM25	transmembrane protein 25
207	70	hsa-miR-370-3p	RNF169	ring finger protein 169
208	70	hsa-miR-370-3p	ARCN1	archain 1
209	70	hsa-miR-370-3p	SLC9A4	solute carrier family 9 member A4
210	70	hsa-miR-370-3p	TAOK1	TAO kinase 1
211	70	hsa-miR-370-3p	RHOBTB2	Rho related BTB domain containing 2
212	70	hsa-miR-370-3p	SORT1	sortilin 1
213	70	hsa-miR-370-3p	RETSAT	retinol saturase
214	70	hsa-miR-370-3p	TPGS2	tubulin polyglutamylase complex subunit 2
215	70	hsa-miR-370-3p	PBX1	PBX homeobox 1
216	70	hsa-miR-370-3p	BMF	Bcl2 modifying factor
217	70	hsa-miR-370-3p	AMIGO2	adhesion molecule with Ig like domain 2

218	70	hsa-miR-370-3p	KCNJ11	potassium voltage-gated channel subfamily J member 11
219	70	hsa-miR-370-3p	F2RL2	coagulation factor II thrombin receptor like 2
220	69	hsa-miR-370-3p	SPANXN1	SPANX family member N1
221	69	hsa-miR-370-3p	NSUN4	NOP2/Sun RNA methyltransferase family member 4
222	69	hsa-miR-370-3p	PACS1	phosphofurin acidic cluster sorting protein 1
223	69	hsa-miR-370-3p	SLC46A1	solute carrier family 46 member 1
224	69	hsa-miR-370-3p	GNAO1	G protein subunit alpha o1
225	69	hsa-miR-370-3p	EGLN3	egl-9 family hypoxia inducible factor 3
226	69	hsa-miR-370-3p	PRDM15	PR/SET domain 15
227	69	hsa-miR-370-3p	SMO	smoothened, frizzled class receptor
228	69	hsa-miR-370-3p	FAM3A	family with sequence similarity 3 member A
229	69	hsa-miR-370-3p	NKTR	natural killer cell triggering receptor
230	69	hsa-miR-370-3p	CDHR1	cadherin related family member 1
231	68	hsa-miR-370-3p	ESRP1	epithelial splicing regulatory protein 1
232	68	hsa-miR-370-3p	ERVFRD-1	endogenous retrovirus group FRD member 1, envelope
233	68	hsa-miR-370-3p	WWP2	WW domain containing E3 ubiquitin protein ligase 2
234	68	hsa-miR-370-3p	N4BP2L1	NEDD4 binding protein 2 like 1
235	68	hsa-miR-370-3p	RUNX1T1	RUNX1 translocation partner 1
236	68	hsa-miR-370-3p	ARNT	aryl hydrocarbon receptor nuclear translocator
237	68	hsa-miR-370-3p	ZFP90	ZFP90 zinc finger protein
238	68	hsa-miR-370-3p	SHE	Src homology 2 domain containing E
239	68	hsa-miR-370-3p	KIF1B	kinesin family member 1B
240	68	hsa-miR-370-3p	SYNDIG1L	synapse differentiation inducing 1 like
241	68	hsa-miR-370-3p	PATJ	PATJ, crumbs cell polarity complex component
242	68	hsa-miR-370-3p	UFD1	ubiquitin recognition factor in ER associated degradation 1
243	68	hsa-miR-370-3p	MYRF	myelin regulatory factor
244	68	hsa-miR-370-3p	SYNPO2	synaptopodin 2
245	68	hsa-miR-370-3p	AP5Z1	adaptor related protein complex 5 subunit zeta 1
246	67	hsa-miR-370-3p	HIF1AN	hypoxia inducible factor 1 subunit alpha inhibitor
247	67	hsa-miR-370-3p	AKAP13	A-kinase anchoring protein 13
248	67	hsa-miR-370-3p	BCCIP	BRCA2 and CDKN1A interacting protein
249	67	hsa-miR-370-3p	LIN28A	lin-28 homolog A
250	67	hsa-miR-370-3p	KDR	kinase insert domain receptor
251	67	hsa-miR-370-3p	RANBP6	RAN binding protein 6
252	66	hsa-miR-370-3p	KRTAP10-11	keratin associated protein 10-11
253	66	hsa-miR-370-3p	PAQR4	progestin and adipoQ receptor family member 4
254	66	hsa-miR-370-3p	ATP8B4	ATPase phospholipid transporting 8B4 (putative)
255	66	hsa-miR-370-3p	MAPK8	mitogen-activated protein kinase 8
256	66	hsa-miR-370-3p	TGFBR2	transforming growth factor beta receptor 2
257	66	hsa-miR-370-3p	NT5C1B-RDH14	NT5C1B-RDH14 readthrough
258	66	hsa-miR-370-3p	RAB3B	RAB3B, member RAS oncogene family
259	66	hsa-miR-370-3p	PLEKHA6	pleckstrin homology domain containing A6
260	66	hsa-miR-370-3p	MMAB	metabolism of cobalamin associated B
261	66	hsa-miR-370-3p	BCL2L11	BCL2 like 11
262	66	hsa-miR-370-3p	RBP1	retinol binding protein 1
263	66	hsa-miR-370-3p	FAM3B	family with sequence similarity 3 member B
264	66	hsa-miR-370-3p	EYA3	EYA transcriptional coactivator and phosphatase 3
265	66	hsa-miR-370-3p	FRMD4B	FERM domain containing 4B
266	65	hsa-miR-370-3p	SGCD	sarcoglycan delta
267	65	hsa-miR-370-3p	HSP90AB1	heat shock protein 90 alpha family class B member 1
268	65	hsa-miR-370-3p	HDX	highly divergent homeobox
269	65	hsa-miR-370-3p	FAM102A	family with sequence similarity 102 member A
270	65	hsa-miR-370-3p	PIAS4	protein inhibitor of activated STAT 4
271	65	hsa-miR-370-3p	DCAF10	DDB1 and CUL4 associated factor 10

272	65	hsa-miR-370-3p	UNC5C	unc-5 netrin receptor C
273	65	hsa-miR-370-3p	NEK9	NIMA related kinase 9
274	65	hsa-miR-370-3p	IGF2BP1	insulin like growth factor 2 mRNA binding protein 1
275	65	hsa-miR-370-3p	WASF2	WAS protein family member 2
276	65	hsa-miR-370-3p	NUDT2	nudix hydrolase 2
277	65	hsa-miR-370-3p	ATAT1	alpha tubulin acetyltransferase 1
278	65	hsa-miR-370-3p	TRIM55	tripartite motif containing 55
279	65	hsa-miR-370-3p	MEGF9	multiple EGF like domains 9
280	65	hsa-miR-370-3p	POM121	POM121 transmembrane nucleoporin
281	65	hsa-miR-370-3p	FNDC7	fibronectin type III domain containing 7
282	64	hsa-miR-370-3p	EEF2K	eukaryotic elongation factor 2 kinase
283	64	hsa-miR-370-3p	ARHGEF37	Rho guanine nucleotide exchange factor 37
284	64	hsa-miR-370-3p	STUM	stum, mechanosensory transduction mediator homolog
285	64	hsa-miR-370-3p	CCDC32	coiled-coil domain containing 32
286	64	hsa-miR-370-3p	NR6A1	nuclear receptor subfamily 6 group A member 1
287	64	hsa-miR-370-3p	NXF1	nuclear RNA export factor 1
288	64	hsa-miR-370-3p	KCNQ5	potassium voltage-gated channel subfamily Q member 5
289	64	hsa-miR-370-3p	UBXN4	UBX domain protein 4
290	64	hsa-miR-370-3p	CHP2	calcineurin like EF-hand protein 2
291	64	hsa-miR-370-3p	NDUFB4	NADH:ubiquinone oxidoreductase subunit B4
292	64	hsa-miR-370-3p	KCNK10	potassium two pore domain channel subfamily K member 10
293	64	hsa-miR-370-3p	WDR82	WD repeat domain 82
294	64	hsa-miR-370-3p	MPV17L2	MPV17 mitochondrial inner membrane protein like 2
295	63	hsa-miR-370-3p	TMEM86A	transmembrane protein 86A
296	63	hsa-miR-370-3p	ADCY6	adenylate cyclase 6
297	63	hsa-miR-370-3p	PLXNA4	plexin A4
298	63	hsa-miR-370-3p	GPATCH2L	G-patch domain containing 2 like
299	63	hsa-miR-370-3p	KIAA0355	KIAA0355
300	63	hsa-miR-370-3p	G6PC2	glucose-6-phosphatase catalytic subunit 2
301	63	hsa-miR-370-3p	PIK3CG	phosphatidylinositol-4,5-bisphosphate 3-kinase catalytic subunit gamma
302	63	hsa-miR-370-3p	CDH13	cadherin 13
303	63	hsa-miR-370-3p	FOSL2	FOS like 2, AP-1 transcription factor subunit
304	63	hsa-miR-370-3p	TRIM59	tripartite motif containing 59
305	63	hsa-miR-370-3p	FBXO40	F-box protein 40
306	63	hsa-miR-370-3p	SH3BP2	SH3 domain binding protein 2
307	63	hsa-miR-370-3p	TMUB2	transmembrane and ubiquitin like domain containing 2
308	63	hsa-miR-370-3p	ACSBG2	acyl-CoA synthetase bubblegum family member 2
309	63	hsa-miR-370-3p	BSDC1	BSD domain containing 1
310	63	hsa-miR-370-3p	SAP30BP	SAP30 binding protein
311	63	hsa-miR-370-3p	SLC10A7	solute carrier family 10 member 7
312	63	hsa-miR-370-3p	CREB3L2	cAMP responsive element binding protein 3 like 2
313	63	hsa-miR-370-3p	IFNAR1	interferon alpha and beta receptor subunit 1
314	62	hsa-miR-370-3p	SNX8	sorting nexin 8
315	62	hsa-miR-370-3p	RAB6B	RAB6B, member RAS oncogene family
316	62	hsa-miR-370-3p	RPRD1A	regulation of nuclear pre-mRNA domain containing 1A
317	62	hsa-miR-370-3p	AQP4	aquaporin 4
318	62	hsa-miR-370-3p	PRMT2	protein arginine methyltransferase 2
319	62	hsa-miR-370-3p	RAD54L2	RAD54 like 2
320	62	hsa-miR-370-3p	PRPF38A	pre-mRNA processing factor 38A
321	62	hsa-miR-370-3p	LPCAT1	lysophosphatidylcholine acyltransferase 1
322	62	hsa-miR-370-3p	USP35	ubiquitin specific peptidase 35
323	62	hsa-miR-370-3p	SLC9A5	solute carrier family 9 member A5
324	62	hsa-miR-370-3p	GOSR1	golgi SNAP receptor complex member 1
325	62	hsa-miR-370-3p	ZNF398	zinc finger protein 398

326	62	hsa-miR-370-3p	CCNE2	cyclin E2
327	62	hsa-miR-370-3p	RBBP8NL	RBBP8 N-terminal like
328	62	hsa-miR-370-3p	ZHX3	zinc fingers and homeoboxes 3
329	62	hsa-miR-370-3p	FGF11	fibroblast growth factor 11
330	62	hsa-miR-370-3p	ATP9A	ATPase phospholipid transporting 9A (putative)
331	61	hsa-miR-370-3p	ZNF75A	zinc finger protein 75a
332	61	hsa-miR-370-3p	TANC1	tetratricopeptide repeat, ankyrin repeat and coiled-coil containing 1
333	61	hsa-miR-370-3p	CRLF1	cytokine receptor like factor 1
334	61	hsa-miR-370-3p	BSPH1	binder of sperm protein homolog 1
335	61	hsa-miR-370-3p	TNS1	tensin 1
336	61	hsa-miR-370-3p	CRYAA2	crystallin alpha A2
337	61	hsa-miR-370-3p	TBC1D16	TBC1 domain family member 16
338	61	hsa-miR-370-3p	CACNG8	calcium voltage-gated channel auxiliary subunit gamma 8
339	61	hsa-miR-370-3p	MDGA1	MAM domain containing glycosylphosphatidylinositol anchor 1
340	61	hsa-miR-370-3p	KCNK3	potassium two pore domain channel subfamily K member 3
341	61	hsa-miR-370-3p	ARF3	ADP ribosylation factor 3
342	61	hsa-miR-370-3p	SETD7	SET domain containing 7, histone lysine methyltransferase
343	61	hsa-miR-370-3p	AAK1	AP2 associated kinase 1
344	61	hsa-miR-370-3p	TMEM192	transmembrane protein 192
345	61	hsa-miR-370-3p	MLC1	megalencephalic leukoencephalopathy with subcortical cysts 1
346	61	hsa-miR-370-3p	CNTN5	contactin 5
347	60	hsa-miR-370-3p	LMOD1	leiomodin 1
348	60	hsa-miR-370-3p	TTYH3	tweety family member 3
349	60	hsa-miR-370-3p	BAALC	BAALC, MAP3K1 and KLF4 binding
350	60	hsa-miR-370-3p	WNT9B	Wnt family member 9B
351	60	hsa-miR-370-3p	C6orf106	chromosome 6 open reading frame 106
352	60	hsa-miR-370-3p	ILF3	interleukin enhancer binding factor 3
353	60	hsa-miR-370-3p	SPATA2	spermatogenesis associated 2
354	60	hsa-miR-370-3p	SSU72	SSU72 homolog, RNA polymerase II CTD phosphatase
355	60	hsa-miR-370-3p	YPEL1	yippee like 1
356	60	hsa-miR-370-3p	GPR174	G protein-coupled receptor 174
357	60	hsa-miR-370-3p	ARPIN	actin related protein 2/3 complex inhibitor
358	60	hsa-miR-370-3p	IPO9	importin 9
359	60	hsa-miR-370-3p	PRRX1	paired related homeobox 1
360	60	hsa-miR-370-3p	PRRC2B	proline rich coiled-coil 2B
361	59	hsa-miR-370-3p	FBXO22	F-box protein 22
362	59	hsa-miR-370-3p	ATRN	attractin
363	59	hsa-miR-370-3p	TRAM2	translocation associated membrane protein 2
364	59	hsa-miR-370-3p	FRAS1	Fraser extracellular matrix complex subunit 1
365	59	hsa-miR-370-3p	KLHL21	kelch like family member 21
366	59	hsa-miR-370-3p	PALD1	phosphatase domain containing paladin 1
367	59	hsa-miR-370-3p	TMEM231	transmembrane protein 231
368	59	hsa-miR-370-3p	CD9	CD9 molecule
369	59	hsa-miR-370-3p	TMEM127	transmembrane protein 127
370	59	hsa-miR-370-3p	C17orf58	chromosome 17 open reading frame 58
371	59	hsa-miR-370-3p	C1QTNF8	C1q and TNF related 8
372	59	hsa-miR-370-3p	NCDN	neurochondrin
373	59	hsa-miR-370-3p	PLCH2	phospholipase C eta 2
374	59	hsa-miR-370-3p	FHL1	four and a half LIM domains 1
375	59	hsa-miR-370-3p	NXPH3	neurexophilin 3
376	59	hsa-miR-370-3p	CRYAA	crystallin alpha A
377	59	hsa-miR-370-3p	MR1	major histocompatibility complex, class I-related
378	59	hsa-miR-370-3p	ZNF70	zinc finger protein 70

379	59	hsa-miR-370-3p	TET3	tet methylcytosine dioxygenase 3
380	59	hsa-miR-370-3p	KLHL3	kelch like family member 3
381	59	hsa-miR-370-3p	UBXN7	UBX domain protein 7
382	59	hsa-miR-370-3p	GABBR2	gamma-aminobutyric acid type B receptor subunit 2
383	59	hsa-miR-370-3p	ORC5	origin recognition complex subunit 5
384	59	hsa-miR-370-3p	BAZ1B	bromodomain adjacent to zinc finger domain 1B
385	59	hsa-miR-370-3p	CA12	carbonic anhydrase 12
386	59	hsa-miR-370-3p	CNBD2	cyclic nucleotide binding domain containing 2
387	59	hsa-miR-370-3p	AKAP10	A-kinase anchoring protein 10
388	59	hsa-miR-370-3p	CDC25B	cell division cycle 25B
389	59	hsa-miR-370-3p	RAB11FIP3	RAB11 family interacting protein 3
390	59	hsa-miR-370-3p	RAB11A	RAB11A, member RAS oncogene family
391	59	hsa-miR-370-3p	UBE2L3	ubiquitin conjugating enzyme E2 L3
392	58	hsa-miR-370-3p	MAP3K9	mitogen-activated protein kinase kinase kinase 9
393	58	hsa-miR-370-3p	SNAP47	synaptosome associated protein 47
394	58	hsa-miR-370-3p	ITFG1	integrin alpha FG-GAP repeat containing 1
395	58	hsa-miR-370-3p	UCP3	uncoupling protein 3
396	58	hsa-miR-370-3p	NOTCH2NLA	notch 2 N-terminal like A
397	58	hsa-miR-370-3p	USF3	upstream transcription factor family member 3
398	58	hsa-miR-370-3p	RPS6KA6	ribosomal protein S6 kinase A6
399	58	hsa-miR-370-3p	FAM43B	family with sequence similarity 43 member B
400	58	hsa-miR-370-3p	ST3GAL4	ST3 beta-galactoside alpha-2,3-sialyltransferase 4
401	58	hsa-miR-370-3p	KCTD16	potassium channel tetramerization domain containing 16
402	58	hsa-miR-370-3p	EIF4E	eukaryotic translation initiation factor 4E
403	58	hsa-miR-370-3p	COL4A3	collagen type IV alpha 3 chain
404	58	hsa-miR-370-3p	APOL3	apolipoprotein L3
405	58	hsa-miR-370-3p	C2	complement C2
406	58	hsa-miR-370-3p	ADGRL3	adhesion G protein-coupled receptor L3
407	58	hsa-miR-370-3p	GRAMD1B	GRAM domain containing 1B
408	58	hsa-miR-370-3p	TMEM144	transmembrane protein 144
409	58	hsa-miR-370-3p	KATNAL1	katanin catalytic subunit A1 like 1
410	58	hsa-miR-370-3p	XYLT1	xylosyltransferase 1
411	58	hsa-miR-370-3p	KCNQ4	potassium voltage-gated channel subfamily Q member 4
412	58	hsa-miR-370-3p	SBSPON	somatomedin B and thrombospondin type 1 domain containing
413	58	hsa-miR-370-3p	ZSCAN2	zinc finger and SCAN domain containing 2
414	57	hsa-miR-370-3p	PIGH	phosphatidylinositol glycan anchor biosynthesis class H
415	57	hsa-miR-370-3p	HIPK2	homeodomain interacting protein kinase 2
416	57	hsa-miR-370-3p	MAML1	mastermind like transcriptional coactivator 1
417	57	hsa-miR-370-3p	SMARCD1	SWI/SNF related, matrix associated, actin dependent regulator of chromatin, subfamily d, member 1
418	57	hsa-miR-370-3p	ATP7B	ATPase copper transporting beta
419	57	hsa-miR-370-3p	MEIS1	Meis homeobox 1
420	57	hsa-miR-370-3p	MAP4	microtubule associated protein 4
421	57	hsa-miR-370-3p	MAPK13	mitogen-activated protein kinase 13
422	57	hsa-miR-370-3p	SIX1	SIX homeobox 1
423	57	hsa-miR-370-3p	FAM149B1	family with sequence similarity 149 member B1
424	57	hsa-miR-370-3p	BFAR	bifunctional apoptosis regulator
425	57	hsa-miR-370-3p	RNF170	ring finger protein 170
426	57	hsa-miR-370-3p	CLVS2	clavesin 2
427	57	hsa-miR-370-3p	DGKZ	diacylglycerol kinase zeta
428	56	hsa-miR-370-3p	EID1	EP300 interacting inhibitor of differentiation 1
429	56	hsa-miR-370-3p	ELFN2	extracellular leucine rich repeat and fibronectin type III domain containing 2
430	56	hsa-miR-370-3p	HSPBAP1	HSPB1 associated protein 1
431	56	hsa-miR-370-3p	MRAS	muscle RAS oncogene homolog

432	56	hsa-miR-370-3p	PPP1R16B	protein phosphatase 1 regulatory subunit 16B
433	56	hsa-miR-370-3p	PDXK	pyridoxal kinase
434	56	hsa-miR-370-3p	CAVIN1	caveolae associated protein 1
435	56	hsa-miR-370-3p	CLASP2	cytoplasmic linker associated protein 2
436	56	hsa-miR-370-3p	LRTM2	leucine rich repeats and transmembrane domains 2
437	56	hsa-miR-370-3p	RAB11FIP4	RAB11 family interacting protein 4
438	56	hsa-miR-370-3p	KRT80	keratin 80
439	56	hsa-miR-370-3p	ATOH8	atonal bHLH transcription factor 8
440	56	hsa-miR-370-3p	CLIC6	chloride intracellular channel 6
441	56	hsa-miR-370-3p	DNAJB1	DnaJ heat shock protein family (Hsp40) member B1
442	56	hsa-miR-370-3p	PLA2G5	phospholipase A2 group V
443	56	hsa-miR-370-3p	IL5RA	interleukin 5 receptor subunit alpha
444	56	hsa-miR-370-3p	HECTD4	HECT domain E3 ubiquitin protein ligase 4
445	56	hsa-miR-370-3p	ECE1	endothelin converting enzyme 1
446	56	hsa-miR-370-3p	GRIK3	glutamate ionotropic receptor kainate type subunit 3
447	56	hsa-miR-370-3p	FOXRED2	FAD dependent oxidoreductase domain containing 2
448	56	hsa-miR-370-3p	ATPAF2	ATP synthase mitochondrial F1 complex assembly factor 2
449	56	hsa-miR-370-3p	GBX2	gastrulation brain homeobox 2
450	56	hsa-miR-370-3p	VCPIP1	valosin containing protein interacting protein 1
451	56	hsa-miR-370-3p	SLC6A6	solute carrier family 6 member 6
452	55	hsa-miR-370-3p	FCRL2	Fc receptor like 2
453	55	hsa-miR-370-3p	CAMK1D	calcium/calmodulin dependent protein kinase ID
454	55	hsa-miR-370-3p	BRF1	BRF1, RNA polymerase III transcription initiation factor subunit
455	55	hsa-miR-370-3p	DCLRE1B	DNA cross-link repair 1B
456	55	hsa-miR-370-3p	SIDT2	SID1 transmembrane family member 2
457	55	hsa-miR-370-3p	JMY	junction mediating and regulatory protein, p53 cofactor
458	55	hsa-miR-370-3p	SELENOI	selenoprotein I
459	55	hsa-miR-370-3p	SLC39A8	solute carrier family 39 member 8
460	55	hsa-miR-370-3p	C18orf25	chromosome 18 open reading frame 25
461	55	hsa-miR-370-3p	CMTR1	cap methyltransferase 1
462	55	hsa-miR-370-3p	RAB15	RAB15, member RAS oncogene family
463	55	hsa-miR-370-3p	TMEM47	transmembrane protein 47
464	55	hsa-miR-370-3p	CAB39	calcium binding protein 39
465	55	hsa-miR-370-3p	EFNB3	ephrin B3
466	55	hsa-miR-370-3p	TECPR2	tectonin beta-propeller repeat containing 2
467	55	hsa-miR-370-3p	PRR5L	proline rich 5 like
468	55	hsa-miR-370-3p	IQCE	IQ motif containing E
469	55	hsa-miR-370-3p	PTPN14	protein tyrosine phosphatase, non-receptor type 14
470	55	hsa-miR-370-3p	ZBTB39	zinc finger and BTB domain containing 39
471	55	hsa-miR-370-3p	IGF2R	insulin like growth factor 2 receptor
472	54	hsa-miR-370-3p	RSAD1	radical S-adenosyl methionine domain containing 1
473	54	hsa-miR-370-3p	CYB5B	cytochrome b5 type B
474	54	hsa-miR-370-3p	DDX3X	DEAD-box helicase 3 X-linked
475	54	hsa-miR-370-3p	FOXO4	forkhead box O4
476	54	hsa-miR-370-3p	DLD	dihydrolipoamide dehydrogenase
477	54	hsa-miR-370-3p	NKAPL	NFKB activating protein like
478	54	hsa-miR-370-3p	BICDL1	BICD family like cargo adaptor 1
479	54	hsa-miR-370-3p	RBPJ	recombination signal binding protein for immunoglobulin kappa J region
480	54	hsa-miR-370-3p	FDFT1	farnesyl-diphosphate farnesyltransferase 1
481	54	hsa-miR-370-3p	FAM168B	family with sequence similarity 168 member B
482	54	hsa-miR-370-3p	SH3D19	SH3 domain containing 19
483	54	hsa-miR-370-3p	ZBTB7C	zinc finger and BTB domain containing 7C
484	54	hsa-miR-370-3p	INPP5A	inositol polyphosphate-5-phosphatase A
485	54	hsa-miR-370-3p	ESS2	ess-2 splicing factor homolog

486	54	hsa-miR-370-3p	ARID3B	AT-rich interaction domain 3B
487	54	hsa-miR-370-3p	OASL	2'-5'-oligoadenylate synthetase like
488	54	hsa-miR-370-3p	CYP2U1	cytochrome P450 family 2 subfamily U member 1
489	53	hsa-miR-370-3p	TBC1D2B	TBC1 domain family member 2B
490	53	hsa-miR-370-3p	DRD5	dopamine receptor D5
491	53	hsa-miR-370-3p	SHROOM3	shroom family member 3
492	53	hsa-miR-370-3p	BBS12	Bardet-Biedl syndrome 12
493	53	hsa-miR-370-3p	PCM1	pericentriolar material 1
494	53	hsa-miR-370-3p	IFITM10	interferon induced transmembrane protein 10
495	53	hsa-miR-370-3p	ELP5	elongator acetyltransferase complex subunit 5
496	53	hsa-miR-370-3p	CANX	calnexin
497	53	hsa-miR-370-3p	LYNX1	Ly6/neurotoxin 1
498	53	hsa-miR-370-3p	LPP	LIM domain containing preferred translocation partner in lipoma
499	53	hsa-miR-370-3p	FBRS	fibrosin
500	53	hsa-miR-370-3p	AURKA	aurora kinase A
501	53	hsa-miR-370-3p	ZNF207	zinc finger protein 207
502	53	hsa-miR-370-3p	CST2	cystatin SA
503	53	hsa-miR-370-3p	SLC25A10	solute carrier family 25 member 10
504	53	hsa-miR-370-3p	CMTM3	CKLF like MARVEL transmembrane domain containing 3
505	53	hsa-miR-370-3p	ADAM19	ADAM metallopeptidase domain 19
506	53	hsa-miR-370-3p	MAP2K7	mitogen-activated protein kinase kinase 7
507	53	hsa-miR-370-3p	MTSS1	MTSS1, I-BAR domain containing
508	53	hsa-miR-370-3p	ZNF746	zinc finger protein 746
509	53	hsa-miR-370-3p	PCSK6	proprotein convertase subtilisin/kexin type 6
510	53	hsa-miR-370-3p	BPIFB4	BPI fold containing family B member 4
511	53	hsa-miR-370-3p	RASSF1	Ras association domain family member 1
512	53	hsa-miR-370-3p	SNRNP25	small nuclear ribonucleoprotein U11/U12 subunit 25
513	53	hsa-miR-370-3p	PPM1F	protein phosphatase, Mg2+/Mn2+ dependent 1F
514	53	hsa-miR-370-3p	HYDIN	HYDIN, axonemal central pair apparatus protein
515	53	hsa-miR-370-3p	TMEM132D	transmembrane protein 132D
516	53	hsa-miR-370-3p	SEC14L2	SEC14 like lipid binding 2
517	53	hsa-miR-370-3p	SLC25A24	solute carrier family 25 member 24
518	53	hsa-miR-370-3p	HOXB13	homeobox B13
519	52	hsa-miR-370-3p	NSL1	NSL1, MIS12 kinetochore complex component
520	52	hsa-miR-370-3p	GHDC	GH3 domain containing
521	52	hsa-miR-370-3p	DERL2	derlin 2
522	52	hsa-miR-370-3p	MAPT	microtubule associated protein tau
523	52	hsa-miR-370-3p	PDHB	pyruvate dehydrogenase E1 beta subunit
524	52	hsa-miR-370-3p	ADAM9	ADAM metallopeptidase domain 9
525	52	hsa-miR-370-3p	ANKRD54	ankyrin repeat domain 54
526	52	hsa-miR-370-3p	ARHGEF12	Rho guanine nucleotide exchange factor 12
527	52	hsa-miR-370-3p	MECP2	methyl-CpG binding protein 2
528	52	hsa-miR-370-3p	SLAMF8	SLAM family member 8
529	52	hsa-miR-370-3p	CENPO	centromere protein O
530	52	hsa-miR-370-3p	IL16	interleukin 16
531	52	hsa-miR-370-3p	AMFR	autocrine motility factor receptor
532	52	hsa-miR-370-3p	LAMC3	laminin subunit gamma 3
533	52	hsa-miR-370-3p	KCTD15	potassium channel tetramerization domain containing 15
534	52	hsa-miR-370-3p	IL6ST	interleukin 6 signal transducer
535	52	hsa-miR-370-3p	BAHD1	bromo adjacent homology domain containing 1
536	52	hsa-miR-370-3p	ULK1	unc-51 like autophagy activating kinase 1
537	52	hsa-miR-370-3p	POFUT1	protein O-fucosyltransferase 1
538	52	hsa-miR-370-3p	TBCK	TBC1 domain containing kinase
539	52	hsa-miR-370-3p	HPS5	HPS5, biogenesis of lysosomal organelles complex 2 subunit 2

540	52	hsa-miR-370-3p	FBXO32	F-box protein 32
541	52	hsa-miR-370-3p	ZNF148	zinc finger protein 148
542	52	hsa-miR-370-3p	NCAM1	neural cell adhesion molecule 1
543	52	hsa-miR-370-3p	ABLIM2	actin binding LIM protein family member 2
544	52	hsa-miR-370-3p	PHF19	PHD finger protein 19
545	52	hsa-miR-370-3p	SLC16A2	solute carrier family 16 member 2
546	52	hsa-miR-370-3p	EPHA10	EPH receptor A10
547	52	hsa-miR-370-3p	REEP5	receptor accessory protein 5
548	51	hsa-miR-370-3p	SLC6A17	solute carrier family 6 member 17
549	51	hsa-miR-370-3p	CCDC9B	coiled-coil domain containing 9B
550	51	hsa-miR-370-3p	COPS4	COP9 signalosome subunit 4
551	51	hsa-miR-370-3p	SULF2	sulfatase 2
552	51	hsa-miR-370-3p	SLC43A2	solute carrier family 43 member 2
553	51	hsa-miR-370-3p	DAAM1	dishevelled associated activator of morphogenesis 1
554	51	hsa-miR-370-3p	PDE7A	phosphodiesterase 7A
555	51	hsa-miR-370-3p	RSAD2	radical S-adenosyl methionine domain containing 2
556	51	hsa-miR-370-3p	ESYT3	extended synaptotagmin 3
557	51	hsa-miR-370-3p	HEG1	heart development protein with EGF like domains 1
558	51	hsa-miR-370-3p	ARSD	arylsulfatase D
559	51	hsa-miR-370-3p	CCL21	C-C motif chemokine ligand 21
560	51	hsa-miR-370-3p	PCDH19	protocadherin 19
561	51	hsa-miR-370-3p	DOK4	docking protein 4
562	51	hsa-miR-370-3p	PANK2	pantothenate kinase 2
563	51	hsa-miR-370-3p	SCML4	Scm polycomb group protein like 4
564	51	hsa-miR-370-3p	ZNF33A	zinc finger protein 33A
565	51	hsa-miR-370-3p	ZFYVE28	zinc finger FYVE-type containing 28
566	51	hsa-miR-370-3p	HMGXB3	HMG-box containing 3
567	51	hsa-miR-370-3p	TMEM72	transmembrane protein 72
568	51	hsa-miR-370-3p	MCFD2	multiple coagulation factor deficiency 2
569	51	hsa-miR-370-3p	DCK	deoxycytidine kinase
570	51	hsa-miR-370-3p	ZNF106	zinc finger protein 106
571	51	hsa-miR-370-3p	ARNT2	aryl hydrocarbon receptor nuclear translocator 2
572	51	hsa-miR-370-3p	EPHX2	epoxide hydrolase 2
573	50	hsa-miR-370-3p	SLC6A14	solute carrier family 6 member 14
574	50	hsa-miR-370-3p	ZNF556	zinc finger protein 556
575	50	hsa-miR-370-3p	RAB7A	RAB7A, member RAS oncogene family
576	50	hsa-miR-370-3p	CHST3	carbohydrate sulfotransferase 3
577	50	hsa-miR-370-3p	ST6GAL1	ST6 beta-galactoside alpha-2,6-sialyltransferase 1
578	50	hsa-miR-370-3p	FARP2	FERM, ARH/RhoGEF and pleckstrin domain protein 2
579	50	hsa-miR-370-3p	C11orf16	chromosome 11 open reading frame 16
580	50	hsa-miR-370-3p	ALAD	aminolevulinate dehydratase
581	50	hsa-miR-370-3p	ZNF316	zinc finger protein 316
582	50	hsa-miR-370-3p	MATR3	matrin 3
583	50	hsa-miR-370-3p	CDH8	cadherin 8
584	50	hsa-miR-370-3p	NTRK2	neurotrophic receptor tyrosine kinase 2
585	50	hsa-miR-370-3p	RASSF4	Ras association domain family member 4
586	50	hsa-miR-370-3p	RTN4RL1	reticulon 4 receptor like 1
587	50	hsa-miR-370-3p	PPM1B	protein phosphatase, Mg2+/Mn2+ dependent 1B
588	50	hsa-miR-370-3p	GRB10	growth factor receptor bound protein 10
589	50	hsa-miR-370-3p	UBTF	upstream binding transcription factor
590	50	hsa-miR-370-3p	MKNK2	MAP kinase interacting serine/threonine kinase 2
591	50	hsa-miR-370-3p	WDR81	WD repeat domain 81
592	50	hsa-miR-370-3p	TBC1D13	TBC1 domain family member 13
593	50	hsa-miR-370-3p	CD200	CD200 molecule
594	50	hsa-miR-370-3p	IRX2	iroquois homeobox 2
595	50	hsa-miR-370-3p	LOC389895	chromosome 16 open reading frame 72-like

596	50	hsa-miR-370-3p	EIF2B1	eukaryotic translation initiation factor 2B subunit alpha
597	50	hsa-miR-370-3p	ZC2HC1C	zinc finger C2HC-type containing 1C
598	50	hsa-miR-370-3p	MFSD1	major facilitator superfamily domain containing 1
599	50	hsa-miR-370-3p	KCNQ2	potassium voltage-gated channel subfamily Q member 2
600	50	hsa-miR-370-3p	P4HA1	prolyl 4-hydroxylase subunit alpha 1
601	50	hsa-miR-370-3p	CNTNAP2	contactin associated protein like 2
602	50	hsa-miR-370-3p	TIGAR	TP53 induced glycolysis regulatory phosphatase
603	50	hsa-miR-370-3p	GLYCTK	glycerate kinase
604	50	hsa-miR-370-3p	RAVER1	ribonucleoprotein, PTB binding 1
605	50	hsa-miR-370-3p	ZFAND5	zinc finger AN1-type containing 5
606	50	hsa-miR-370-3p	UBE2R2	ubiquitin conjugating enzyme E2 R2
607	50	hsa-miR-370-3p	SERTAD2	SERTA domain containing 2
608	50	hsa-miR-370-3p	ODF2	outer dense fiber of sperm tails 2
609	50	hsa-miR-370-3p	BHLHB9	basic helix-loop-helix family member b9
