# Supplementary figures and images for: Circular RNA hsa-circ-0005238 enhances trophoblast migration, invasion and suppresses apoptosis via the miR-370-3p/CDC25B axis
Source: Front Med (Lausanne). 2022 Oct 13;9:943885. doi: 10.3389/fmed.2022.943885 (PMC9606333; doi:10.3389/fmed.2022.943885)

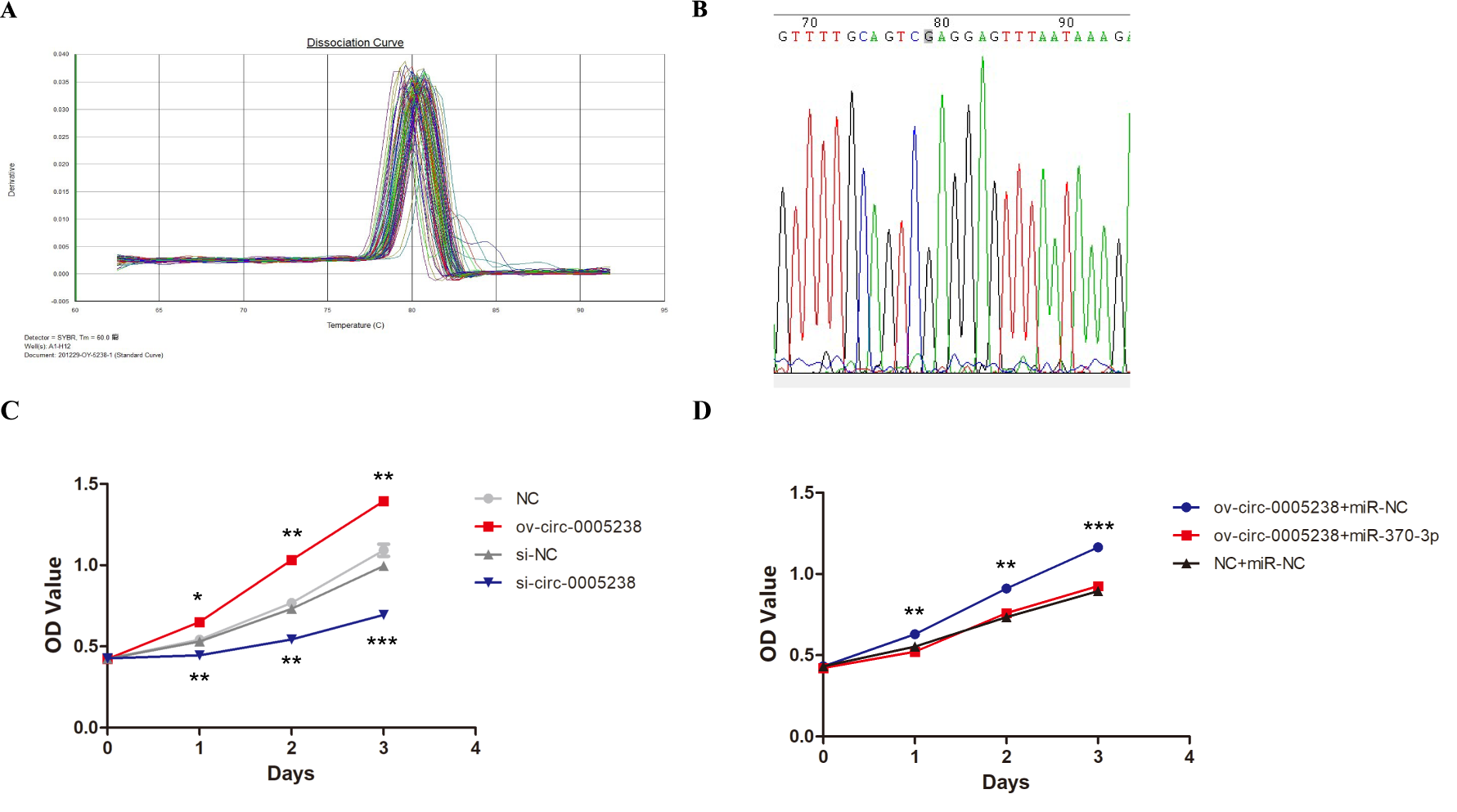

Supplement: Supplementary Figure 1 — (A) Supplementary melting curve analysis of hsa-circ-0005238. (B) Sequencing analysis around the splice junction of hsa-circ-0005238 fragment in RT-qPCR. (C) Cell proliferation results of hsa-circ-0005238 overexpression or knockdown. (D) Cell proliferation results of reverse experiment. *P < 0.05, **P < 0.01, ***P < 0.001. [file Image_1.TIF]

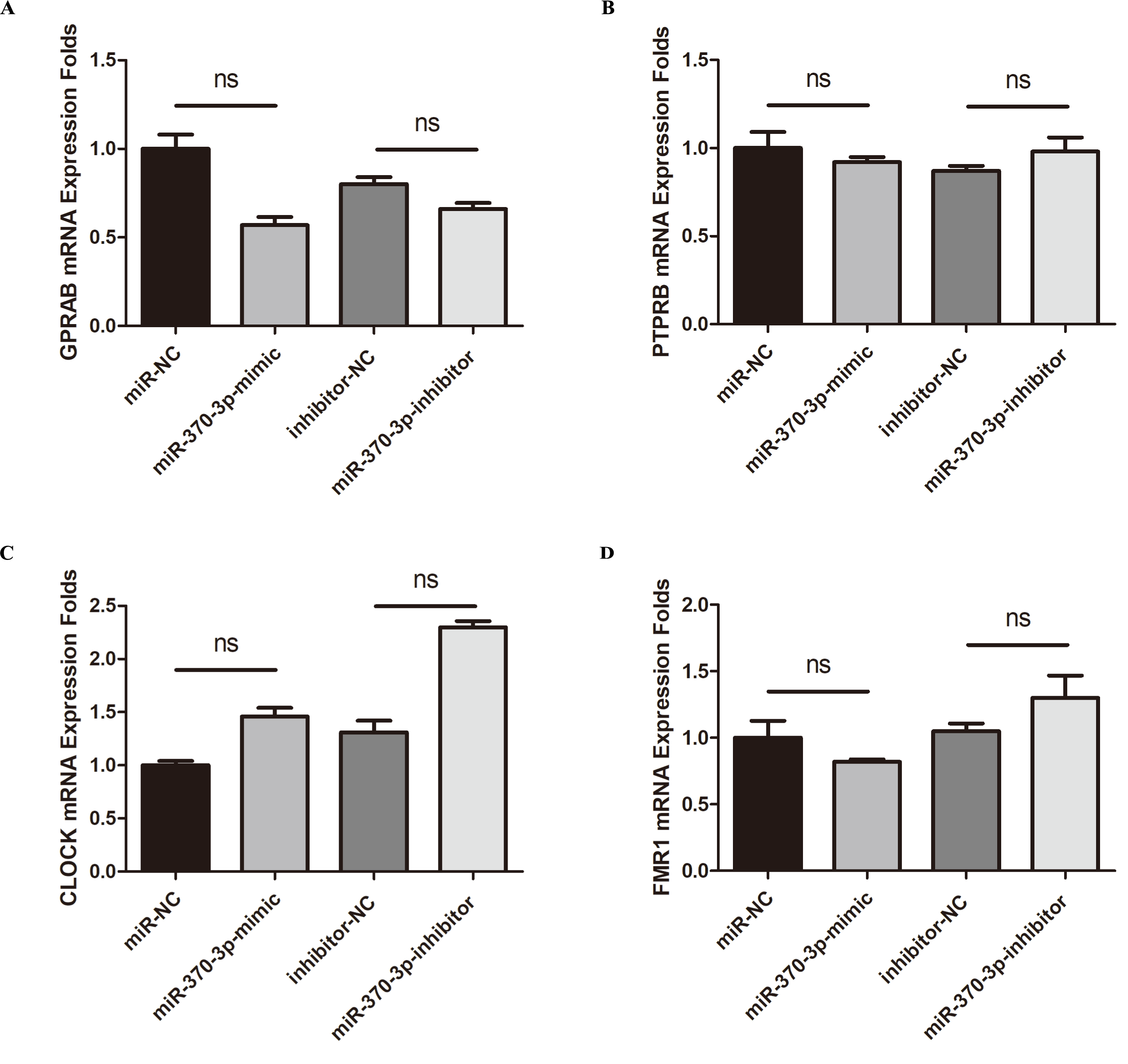

Supplement: Supplementary Figure 2 — Expression of GPRAB, PTPRB, CLOCK, and FMR1 in miR-370-3p-mimic, miR-NC, inhibitor- miR-370-3p, and inhibitor-NC group by RT-qPCR. (A) Expression of GPRAB. (B) Expression of PTPRB. (C) Expression of CLOCK. (D) Expression of FMR1. [file Image_2.TIF]

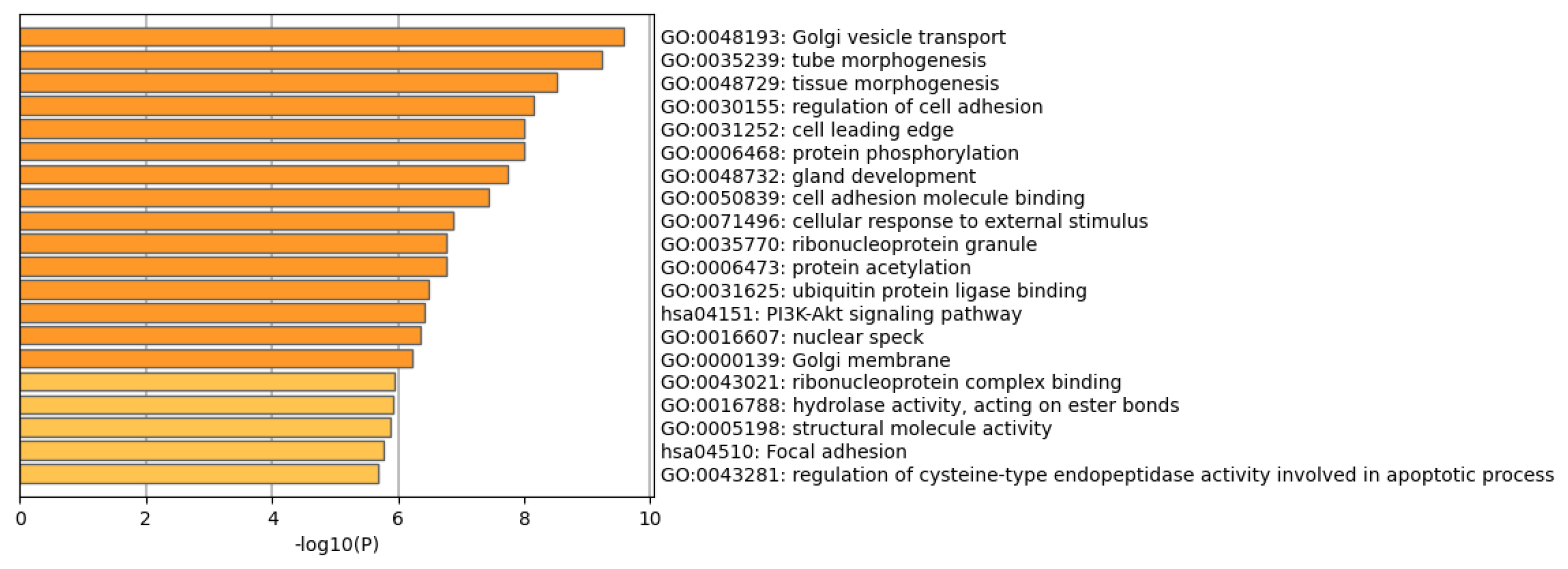

Supplement: Supplementary Figure 3 — Top 20 pathway terms in GO enrichment analysis of hsa-circ-0005238. [file Image_3.TIF]
